# Incomplete tricarboxylic acid cycle and proton gradient in *Pandoravirus massiliensis*: is it still a virus?

**DOI:** 10.1038/s41396-021-01117-3

**Published:** 2021-09-23

**Authors:** Sarah Aherfi, Djamal Brahim Belhaouari, Lucile Pinault, Jean-Pierre Baudoin, Philippe Decloquement, Jonatas Abrahao, Philippe Colson, Anthony Levasseur, David C. Lamb, Eric Chabriere, Didier Raoult, Bernard La Scola

**Affiliations:** 1 Aix Marseille Univ, IRD, MEPHI, Marseille, France; 2 Assistance Publique—Hôpitaux de Marseille (AP-HM), Marseille, France; 3 Institut Hospitalo-Universitaire (IHU)—Méditerranée Infection, Marseille, France; 4 Laboratório de Vírus, Departamento de Microbiologia, Instituto de Ciências Biológicas, Universidade Federal de Minas Gerais, Belo, Horizonte Brazil; 5 Faculty of Health and Life Sciences, Swansea University, Swansea, UK

**Keywords:** Virology, Molecular evolution

## Abstract

The discovery of *Acanthamoeba polyphaga* Mimivirus, the first isolated giant virus of amoeba, challenged the historical hallmarks defining a virus. Giant virion sizes are known to reach up to 2.3 µm, making them visible by optical microscopy. Their large genome sizes of up to 2.5 Mb can encode proteins involved in the translation apparatus. We have investigated possible energy production in *Pandoravirus massiliensis*. Mitochondrial membrane markers allowed for the detection of a membrane potential in purified virions and this was enhanced by a regulator of the tricarboxylic acid cycle but abolished by the use of a depolarizing agent. Bioinformatics was employed to identify enzymes involved in virion proton gradient generation and this approach revealed that eight putative *P. massiliensis* proteins exhibited low sequence identities with known cellular enzymes involved in the universal tricarboxylic acid cycle. Further, all eight viral genes were transcribed during replication. The product of one of these genes, ORF132, was cloned and expressed in *Escherichia coli*, and shown to function as an isocitrate dehydrogenase, a key enzyme of the tricarboxylic acid cycle. Our findings show for the first time that a membrane potential can exist in Pandoraviruses, and this may be related to tricarboxylic acid cycle. The presence of a proton gradient in *P. massiliensis* makes this virus a form of life for which it is legitimate to ask the question “what is a virus?”.

## Introduction

Since the discovery of *Acanthamoeba polyphaga* Mimivirus [1] in 2003, giant viruses of amoeba have consistently challenged the historical definition and classification of viruses [2]. With a virion size larger than 200 nm [1] and encompassing genome sizes greater than 250 kb [3], giant viruses differ from all previously described viruses to date. In 2013, *Pandoravirus salinus*, the first Pandoravirus isolated, broke all genomic viral size records with a genome size of 2.5 Mbp and 1-µm-diameter viral particles [4]. Moreover, Pandoravirus genomes to-date do not harbor any gene(s) encoding capsid protein(s), another hallmark of viral biology [4]. When observed by transmission electron microscopy (TEM), Pandoraviruses may utilize host cellulose production to build tegument [5]. Among later scientific discoveries causing giant viruses to challenge viral definition were the findings of associated virophages, which depicted for the first time a virus being infected by another virus [6]. In 2016, the MIMIVIRE system was identified as a functioning defense mechanism in Mimiviruses against invading virophages [7]. This was the first time that a mechanism for destroying invading foreign DNA, analogous to CRISPR-Cas system in bacteria, was observed to function in a virus. In 2018, the identification of Mimivirus proteins involved in protein translation again challenged another key feature of the definition of viruses [8]. Subsequently, an almost complete set of protein translation apparatus was discovered in *Tupanvirus* and *Klosneuviruses* [9, 10]. Finally, in 2019 it was found that genes encoding multiple and unique cytochromes P450 monooxygenases commonly occur in giant viruses in the *Mimiviridae*, *Pandoraviridae*, and other families in the proposed order Megavirales [11, 12].

Recent data indicate that some giant viruses possess genes that may be involved in metabolic pathways such as fermentation, sphingolipid biosynthesis and nitrogen metabolism [13]. Moreover, tupanviruses harbor a gene coding for citrate synthase [14]. These viral genes are believed to be used to manipulate host metabolic pathways. However, no evidence has emerged to-date suggesting that such viruses use these gene products themselves for their own metabolic needs. As giant viruses have challenged nearly all criteria defining a virus, we decided to test another key hallmark of independent life in viruses: the ability to produce energy. To test this hypothesis, we used the giant virus *Pandoravirus massiliensis*, which we recently isolated [15]. The Pandoravirus family stands uniquely apart from other giant viruses of amoebas because of their huge gene content, with more than 80% of their predicted gene products being ORFans (no homologs in international protein databases). Hence, *P. massiliensis* provides a novel viral system for the discovery of genes with currently unknown functions. In the living world, energy generation is mostly associated with the creation of proton gradients. Thus, we searched for energy gradients in *P. massiliensis*. We were able to observe the presence of a proton gradient in this virus, and surprisingly, it was mainly present in the mature particles. We then searched for genes that could be responsible for this proton gradient. No genes involved in the respiratory chain or with identity to ATP synthase were detected. However, gene products having weak identities with nearly all enzymes of the tricarboxylic acid (TCA) cycle were observed. These viral genes were transcribed together, and the product of at least one gene, isocitrate dehydrogenase (IDH), was functional. These findings argue that *P. massiliensis* is a viral life form for which it is now legitimate to ask the question: “What is a virus?”

## Materials and methods

### *P. massiliensis* immunofluorescence staining using mouse specific polyclonal antibodies

In the aim to avoid any confusion with the staining of the amoeba mitochondria, mouse polyclonal antibodies were generated to specifically stain the isolated *P. massiliensis* mature virions and their replication cycle into amoeba cells. We first immunized a mouse with *P. massiliensis* by the subcutaneous route. After three inoculations, mouse serum containing polyclonal antibodies specific to *P. massiliensis* was collected and adsorbed on uninfected *A. castellanii* lysate to remove nonspecific antibodies targeting amoeba [16]. To permeabilize the cell membranes and saturate the nonspecific binding sites, cells were incubated in fetal calf serum with 1% (v/v) Triton X-100 in phosphate-buffered saline (PBS) for 1 h. Infected amoebas were incubated overnight at 28 °C in a humidified chamber with anti-Pandoravirus antibodies. Subsequently, the samples were washed three times with 0.1% (v/v) Triton X-100 in PBS. Each sample was incubated with fluorescein isothiocyanate (FITC)-conjugated goat anti-mouse IgG (Immunotech, Marseille, France) for 60 min at 28 °C in a humidified chamber and finally washed three times with PBS.

### Assessment of a membrane electrochemical gradient in *P. massiliensis* virions

The membrane electrochemical gradient in *P. massiliensis* was assessed by two dyes: Mitotracker Deep Red 633 (Invitrogen, Carlsbad, California, USA), and tetramethyl rhodamine (TMRM) reagent (Thermo Fisher Scientific). Mitotracker Deep Red 633 and TMRM are cationic fluorescent dyes that are selectively sequestered by mitochondria in live cells based on their charge and that are dependent on the mitochondrial membrane potential for loading.

(i) To assess the membrane electrochemical gradient of *P. massiliensis* during the replication cycle, amoeba were infected with *P. massiliensis* at a multiplicity of infection of ten in IBIDI petri µ-dishes previously coated with poly-L lysine to retain adherent cells. Each sample was dyed with MitoTracker Deep Red 633 for 45 min at 37 °C. The samples were washed twice with PBS to remove the excess fluorescent dye and blocked with acetone at different time points of the viral cycle (2, 4, 6, 8, 10, 12, 14, and 16 h), and then washed three times with PBS. To avoid any further confusion with staining of the amoeba mitochondria with MitoTracker Deep Red 633, immunofluorescence staining using mouse specific polyclonal antibodies targeting *P. massiliensis* inside the amoeba was also undertaken following the protocol described above.

(ii) To assess the membrane electrochemical gradient in the purified *P. massiliensis* virions, both MitoTracker Deep Red 633 and TMRM were used.

Briefly, lysed amoeba infected by *P. massiliensis* were filtered (5 µm) then centrifuged 10 min at 500 × *g*, and cellular debris was discarded. The viral supernatant was centrifuged 20 min at 6800 × *g*, and the pellet was resuspended twice in PAS. The second time, the viral pellet was resuspended in survival buffer. For MitoTracker Deep Red 633 staining, purified viral particles were incubated with the dye for 45 min at 37 °C. Subsequently, the sample was washed twice with PBS to remove the excess fluorescent dye and fixed with acetone. Immunofluorescence staining using mouse specific polyclonal antibodies targeting *Pandoravirus massiliensis* was also carried out.

For TMRM, 1 mL of purified viral particles was incubated with 1 µl of stock solution of TMRM (100 µM) for 30 min at 30 °C in IBIDI petri µ-dishes. In control experiments, sample cultures of *Staphylococcus aureus* were used as positive controls, and viral supernatant from *cowpoxvirus* cultured on Vero (ATCC CCL-81) African green monkey kidney cells [17] was used as negative control.

To ensure the specificity of Mitotracker fixation on virions alone and to avoid misleading observations, in addition to immunofluorescence staining using mouse specific polyclonal antibodies targeting *P. massiliensis*, we also checked for the absence of amoebal structure debris including mitochondria by scanning electron microscopy with the SU5000 device (Hitachi High-Technologies, Tokyo, Japan). Scanning electron microscopy was parallelly carried out on both purified virions and on amoebal isolated mitochondria to exclude any possibility of morphological confusion between these two structures [18].

### Assessment of the effect of the decoupling agent CCCP on viral particles

To determine that fluorescence observed with the two mitochondrial dyes was specifically linked to the existence of an electrochemical gradient throughout the virions tegument, we used carbonyl cyanide m-chlorophenylhydrazone (CCCP) (Sigma-Aldrich C2759; Saint-Louis, Missouri, USA), an inhibitor of oxidative phosphorylation that acts by dissipating the electrochemical gradient induced by the proton concentration. CCCP effect was assessed on *P. massiliensis* virions treated with TMRM. CCCP reagent (100, 200, 300, 400 µM) was directly added in tubes containing ≈10^7^ viral particles/ml. Negative control were *P. massiliensis* virions without CCCP. Samples were incubated at 35 °C overnight and then transferred to petri µ-dishes. Next, 1 µl of a stock solution of TMRM (100 µM) was added and plates incubated 30 min at 30 °C. Images were acquired by confocal microscopy using a Zeiss LSM 800 microscope. To assess if the CCCP could have an impact on the infectiveness of virions, the infectivity of *P. massiliensis* particles was assessed before and after incubation with CCCP by calculating the TCID50 using the method of Reed and Muench [19]. The potential impact of CCCP on viral replication cycle was also assessed by immunofluorescence and qPCR. *A. castellanii* strain Neff cells were inoculated with viral particles previously incubated with 400 µM CCCP and washed three times. As at the end of the replication cycle, no differences were observed, so we focused on early time points of the viral cycle. Infected amoeba were collected 45 min (H0) and 3 h (H3) postinfection. *P. massiliensis* particles spotted on slides were labeled with anti-*P. massiliensis* specific antibodies according to the protocol described above. qPCR was carried out using isolated DNA from the collected cells targeting the DNA polymerase gene of *P. massiliensis* by PCR (forward primer: 5′-ATGGCGCCCGTCTGGAAG; reverse primer: 5′-GGCGCCAAAGTGGTGCGA). qPCR was performed with a LightCycler 480 SYBR Green 1 Master reaction mix (Roche Diagnostics, Mannheim, Germany), following the manufacturer’s temperature program with 60 °C for the primer hybridization and elongation temperature.

### Assessment of the effect of acetyl-CoA on *P. massiliensis* viral particles

To assess if the potential electrochemical gradient observed in virions could be modified, we chose to study the impact of the acetyl-CoA, a well-know regulator of the TCA cycle. Acetyl-CoA (Sigma-Aldrich) was added at concentrations of 0.8 mM, 0.4 mM, 0.2 mM, 0.1 mM, 0.01 mM, and 0.001 mM to 1 mL of the viral suspension of purified particles (≈10^8^ particles/ml). The negative control consisted in viral particles without acetyl-CoA. The samples were incubated at 30 °C for 24 h. After incubation, the samples were transferred into petri µ-dishes and stained with TMRM by following the above-described protocol. Images were acquired using a LSM 800 confocal microscope. Image processing and fluorescence intensity evaluations were conducted using Zen Bleu software.

### Bioinformatics and phylogenetics analyses

The *P. massiliensis* genome was analyzed by BLASTp analyses against the GenBank nr database version 2.10.0+ using an e-value threshold of 1 × 10^−2^. The search for gene products potentially involved in energy metabolism was performed by delta-BLAST analyses against the Conserved Domain Database (CDD) [20, 21]. For some predicted ORFs having hits with low similarity, PSI Blast, HMM searches against PFAM database (http://hmmer.org; HmmerWeb version 2.41.1), HHPRED (version of the database: 20200717) analyses [22] and structure prediction using the PHYRE2v2.0 server were performed [23]. Orthologs in other pandoravirus genomes were searched using the ProteinOrtho tool with a 30% identity percentage threshold and 50% as a coverage percentage threshold [24]. Gene products of all Pandoraviruses were also analyzed by BLASTp against the COG database version 2003 COGs, 2014 update [25, 26]. The viral ORFs harboring a hit against class C of the COG (energy metabolism) with a bitscore >50 were considered significant.

Sequences were aligned with Muscle software [27]. Phylogenetic tree was built by using FastTree v2.1.10 [28] with the maximum likelihood method and formatted with the iTOL v6.1.1 program [29]. The robustness of the groupings was assessed by bootstrap resampling of 1000 replicates and only significant bootstrap values (>70%) were displayed.

### Transcriptome sequencing (RNA-seq) on *P. massiliensis*

The transcriptome of *P. massiliensis* strain BZ81 was assessed as described previously using amoebas infected by *P. massiliensis* [15] as well as freshly released mature viral particles. Mature virions were collected 11 h following amoeba inoculation, passed through 5-µm-pore filters, and centrifuged at 500 × *g* for 10 min to remove all amoeba debris.

### qRT-PCR of suspected *P. massiliensis* TCA cycle genes

Viral DNA was isolated from 200 µL of viral supernatant from *P. massiliensis* culture using the EZ1 tissue kit (Qiagen, France) according to the manufacturer’s recommendations. Before RNA extraction, all samples were incubated with Turbo DNase for 30 min at 37 °C. RNA was extracted using the RNeasy mini kit (Qiagen, France) at different time points of the *P. massiliensis* replication cycle, from H0 (i.e., 45 min after infection of ameba cells by viral particles) until H16 postinfection (release of neo-synthetized virions), according to the manufacturer’s recommendations). Subsequently, three further digestions with Turbo DNase (Invitrogen, United States) were performed for each RNA sample to eliminate DNA contamination. Finally, enzyme inactivation reagent was added and incubated at room temperature for 5 min. The samples were then centrifuged at 10,000 × *g* for 1.5 min. Two PCR systems targeting the *P. massiliensis* DNA polymerase gene (forward primer: 5′-ATGGCGCCCGTCTGGAAG; reverse primer: 5′-GGCGCCAAAGTGGTGCGA) and the housekeeping gene of the RNA polymerase of *A. castellanii* (forward: 5′-ACGAACTTCCGAGAGATGCA; reverse: 5′-CACCTTGACCAGTCCCTTCT) were used to check for genomic DNA contamination.

Nucleotide primers targeting the selected ORFs of the *P. massiliensis* gene sequences were designed using the primer3 tool [30] (Supplementary File 1). RT-qPCR was performed in a one-step reaction using the QuantiTect SYBR Green RT-PCR Kit (QIAgen, France) following the manufacturer’s recommendations. Each experiment was performed in triplicate. The results were considered positive if the cycle threshold obtained in three replicates was less than 35.

### Proteome analysis of *P. massiliensis*

Protein extraction was carried out on purified viral particles and amoebas infected with *P. massiliensis* at different time points of the replication cycle (H0 to H16). Samples were treated as previously described [15, 31]. The final analysis was performed with an internal protein sequence database built primarily with two types of amino acid sequences as follows:

(i) Sequences obtained by translating the whole genome into the six reading frames and then fragmenting the six translation products into 250-amino-acid-long sequences with a sliding step of 30 amino acids, stop codons being replaced by a trypsin cleavage site. Contiguous sequences that were positive for peptide detection were fused and reanalyzed. (ii) Host organism sequences (*A. castellanii* str.Neff, Uniprot, 15235 sequences*)*. The virus sequences thus obtained were finally compared with the ORFs.

### Cloning, expression and purification of predicted *P. massiliensis* TCA cycle enzymes

*P. massiliensis* genes encoding the predicted TCA enzymes (ORFs 132, 181, 206, 577, 595, 762, 864, and 1245) were designed to include a Strep-tag at the N-terminus and optimized for *Escherichia coli* expression. Genes were synthesized by GenScript (Piscataway, NJ, USA) and ligated between the *Nde*I and *Not*I sites of a pET22b(+) plasmid. Competent BL21(DE3) cells grown in autoinducing ZYP-5052 medium were used for expression of the recombinant proteins. To produce each protein, the culture was shaken at 37 °C until an O.D. 600 nm of 0.6 was reached, after which the temperature was lowered to 20 °C for 20 h. Cells were harvested by centrifugation (5000 × *g*, 30 min, 4 °C), and the resulting pellet was resuspended in 50 mM Tris pH 8, 300 mM NaCl and stored at −80 °C overnight. The crude extract was thawed and incubated on ice for 1 h following the addition of lysozyme, DNAse I and phenylmethylsulfonyl fluoride (PMSF) to final concentrations of 0.25 mg/mL, 10 µg/mL and 0.1 mM, respectively. Partially lysed cells were sonicated using a Q700 sonicator system (QSonica), and cell debris was removed following a centrifugation step (12,000 × *g*, 20 min, 4 °C). Proteins were purified with an ÄKTA avant system (GE Healthcare) using strep-tag affinity chromatography (Wash buffer: 50 mM Tris pH 8, 300 mM NaCl and Elution buffer: 50 mM Tris pH 8, 300 mM NaCl, 2.5 mM desthiobiotin) on a 5-mL StrepTrap HP column (GE Healthcare). Recombinant protein expression was confirmed by MALDI-TOF MS analysis of excised gel bands previously isolated by SDS-PAGE. Protein concentrations were measured using a Nanodrop 2000c spectrophotometer (Thermo Scientific).

### Enzymatic activity assay and kinetics

Enzymatic activities of the predicted citrate synthase, aconitase and α-ketoglutarate dehydrogenase were tested using the Citrate Synthase Assay kit, Aconitase Activity Assay kit and α-ketoglutarate Dehydrogenase Activity Colorimetric Assay kit, respectively (Sigma-Aldrich, St. Louis, MS, USA). IDH activity assays were performed using the isocitrate dehydrogenase activity assay kit (Sigma-Aldrich, St. Louis, MS, USA) and monitored with a Synergy HT microplate reader (BioTek, Winooski, VT, USA). Reactions were carried out in duplicate at 37 °C in a 96-well plate containing a final volume of 100 µL in each well. Conversion of the isocitrate to α-ketoglutarate was monitored for 30 min following changes in absorbance at 450 nm, corresponding to the production of NADH. A NADH standard curve was constructed, allowing enzyme quantification and calculation of specific activity. In addition, IDH activity was assessed on mature viral particles purified as described above, and on human IDH (Sigma-Aldrich; St. Louis, MS, USA). Initial velocities were calculated using Gen5.1 software (BioTek), and the obtained mean values were fitted using the Michaelis–Menten equation in Prism 6 (GraphPad Software, San Diego, CA, USA).

### Statistical analysis

Statistical analysis was performed using GraphPad Prism for Windows. Statistical differences were evaluated by one-way ANOVA. Statistical significance was set at *p* < 0.05.

## Results and discussion

### Two mitochondrial dyes, MitoTracker and TMRM, identify a membrane potential in mature *P. massiliensis* virions and during its replication cycle

**Fig. 1:**
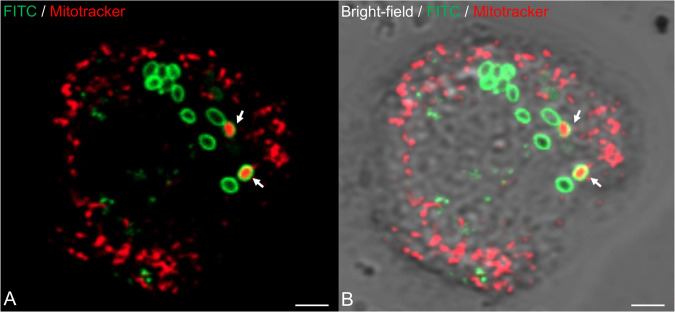
Confocal imaging of amoeba infected by *P. massiliensis* stained with MitoTracker Deep Red (in red) and with specific anti-*P. massiliensis* antibodies (in green). **A** Co-localization of the MitoTracker signal (in red) with virus marked by specific antibodies (FITC) (arrows). **B** Merge of Bright-field, FTIC and MitoTracker fluorescence. The scales bar correspond to 2 µm.

Membrane potential in *P. massiliensis* virions was assessed during the replication cycle in *A*. *castellanii* and mature virions freshly released from amoebas. During the *P. massiliensis* viral cycle in *A. castellanii*, a variable proportion of MitoTracker-labeled viruses was observed (Fig. 1). The specificity of the labeling was ensured by the co-localization of FITC-conjugated anti- *P. massiliensis* antibodies.

**Fig. 2:**
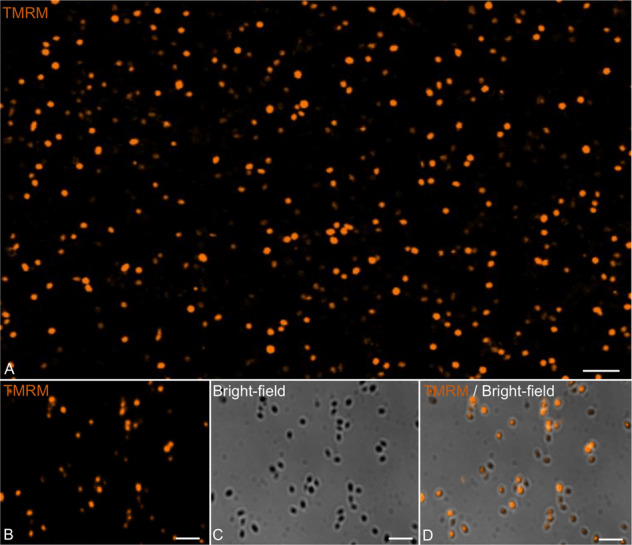
Confocal imaging of TMRM staining of purified *P*. *massiliensis* virions. **A**, **B** Viral mature particles stained with TMRM. **C** Bright-field channel. **D** Merge of Bright-field and TMRM showing the internalization of the TMRM signal in viral particles. The scales bar correspond to 5 µm.

An analogous experiment performed using mature viral particles showed that approximately 20% of the total number of particles were labeled both by anti-*P. massiliensis* specific antibodies and MitoTracker Deep Red 633 (Supplementary Fig. 1). Moreover, the purified viral particles were also labeled by TMRM staining (under the TRITC wavelength (532 nm), with a fluorescent signal (Fig. 2, Supplementary Fig. 2), similar to the results obtained for the *S. aureus* positive control. No fluorescence was observed in the cowpoxvirus negative control experiments. We identified here a virion membrane potential for the first time using two different fluorescent mitochondrial dyes, MitoTracker Deep Red 633 and TMRM, which allowed the detection of a fluorescent signal in mature *P. massiliensis* virions. To note, TMRM has been scientifically acknowledged as the ideal marker to assess mitochondrial membrane potential [32]. It can be argued that the fluorescent signal of the TMRM might be confused with the staining of remaining membrane debris of amoebal mitochondria, since all the enzymes of *A. castellani*, involved in energy production, are localized in mitochondria [33–35]. Therefore, in addition to the use of specific antibodies targeting the virus and after careful separation of Pandoravirus particles from amoebal debris, we checked for the absence of any amoebal structure by 3D analysis (Supplementary Fig. 3). Furthermore, a negative control consisting of purified amoebal mitochondria clearly showed a distinct morphology from those of pandoraviruses virions, thus excluding any misleading conclusions of potential staining by MitoTracker of amoebal debris (Supplementary Fig. 4).

### The depolarizing agent CCCP abolishes the potential membrane of *P. massiliensis*

**Fig. 3:**
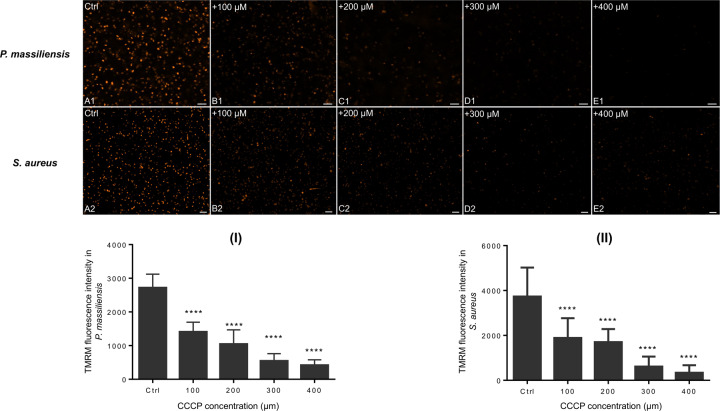
Evaluation of the fluorescence intensity of TMRM after CCCP treatment. **A1–E1** Confocal imaging of TMRM staining following CCCP treatment of *P. massiliensis* particles. **A1** Control experiment using untreated *P. massiliensis* particles. **B1**, **E1**: Confocal imaging of *P. massiliensis* virions treated with different concentrations of CCCP. **A2–E2** Confocal imaging of TMRM staining after CCCP treatment of the positive control (*S. aureus*). **A2** Control experiment showing untreated *S. aureus*. **B2**, **E2** *S. aureus* treatment with a different concentration of CCCP. **I** Estimation of TMRM fluorescence intensity of *P. massiliensis* particles following CCCP treatment. **II** Estimation of TMRM fluorescence intensity of *S. aureus* following CCCP treatment. The scales bar correspond to 5 µm.

*P. massiliensis* particles were incubated with a range of CCCP concentrations: 100, 200, 300, and 400 µM. Analysis of the *S. aureus* positive control revealed the fluorescent signal generated by TMRM decreased significantly (*p* < 0.05) in the presence of all concentrations of CCCP compared with the negative controls (viral particles without CCCP) (Fig. 3).

The titer of viral particles after incubation with CCCP did not show any significant difference compared with the negative control (10^7,2^ TCID_50_/ml). Delta-Ct between the negative control (CCCP untreated samples) and viral particles preincubated with CCCP at 400 µM was 1.85 and 2.57 for the H0 and H3 postinfection time points, respectively. Immunofluorescence revealed a smaller number of labeled viral particles preincubated with CCCP at 400 µM on amoeba cells (1313 and 1613 at H0 and H3, respectively) than the negative control (1658 and 1889 at H0 and H3, respectively), but the difference was not significant (Supplementary Fig. 5). We showed here that the intensity of the membrane potential is abolished following treatment with CCCP, a decoupling agent, confirming the accuracy of the existence of this electrochemical gradient.

We could only observe a lower number of viral particles on amoeba cells infected with virions preincubated with CCCP at H0 and H3 postinfection than in negative controls. This finding suggested that the membrane voltage may be involved in the infection process of amoeba cell, particularly in the early stages of infection. The membrane potential of virions may facilitate the viral DNA release into amoeba host cells. The impact of the osmolarity was previously demonstrated for *Chlorella* viruses for which inhibition of K+ channels prevent the viral DNA release [36] and for bacteriophages for which genome release was suppressed by high external osmotic pressure [37]. Moreover, genes encoding photosynthetic electron transport proteins identified in phages from the *Myoviridae* and *Podoviridae* families, that infect *Prochlorococcus*, a marine cyanobacteria, may help maintaining photosynthesis during viral infection. The phage replication cycle depending on photosynthetic activity, such genes may increase the phage fitness [38]. Furthermore, a metagenomics study carried out on hydrothermal vent plumes and associated deep ocean waters demonstrated that several dsDNA viruses that infect chemolithoautotroph sulfur-oxidizing bacteria, harbor auxiliary metabolic genes for subunits of the reverse dissimilatory sulfite reductase. This enzyme oxidizes elemental sulfur, the most abundant source of chemosynthetic energy in the hydrothermal plumes and the central intermediate in the oxidation of other reduced forms of sulfur used by some sulfur-oxidizing bacteria [39], These viruses may play an essential role in the sulfur cycle in the deep oceans and more widely, in the evolutionary dynamics of a hub in the planetary cycling of sulfur [40]. A host-derived ammonium transporter identified in a phytoplankton virus, which infects *Ostreococcus tauri* green alga, was expressed during viral infection and takes up sources of nitrogen, impacting marine nutrient cycles [41]. *Nitrosopumilus* spindle-shaped viruses, a potential important group of marine viruses that infect ammonia-oxidizing archaea might regulate dynamics, diversity and evolution of host communities and, thus, leading to a reduction in the rate of ammonia oxidation and nitrite reduction, with a global impact on carbon and nitrogen cycling [42]. In resources poor environments, viruses adopt a non lytic mode of replication allowing an adaptive way for survival of viruses [42]. Several other studies have shown that marine viral communities can adapt the diversity of their auxiliary metabolic genes to exploit and master their microbial hosts according to environmental changes, to ensure keeping on an efficient viral replication in nutrient deprived conditions, especially by direct auxiliary metabolic genes-driven metabolic reprogramming [43–46]. Such genes include some involved in the central carbon metabolism, nitrogen and nutrient cyclings, soil organic matter degradation, and consequently play a key role in the global ecosystem [47–51].

### The membrane potential of mature virions is enhanced by low concentrations and decreased by high concentrations of acetyl-CoA

In comparison to negative controls (untreated Pandoravirus particles) (Supplementary Fig. 6.A1), the TMRM fluorescent signal significantly increased in the presence of low concentrations of acetyl-CoA (0.01 mM) (Supplementary Fig. 6.F1.I) (*p* < 0.05) and significantly decreased in the presence of high concentrations of acetyl CoA (0.8 mM, 0.4 mM) (Supplementary Fig. 6.B1.C1.I) (*p* < 0.05). In positive control experiments using *S. aureus*, the TMRM fluorescent signal increased significantly at low concentrations of acetyl-CoA (0.1 mM) (Supplementary Fig. 6.E2.II) (*p* < 0.05) and decreased significantly in the presence of high concentrations of acetyl CoA (0.8 mM, 0.4 mM) (supplementary Fig. 6.B2.C2.II,) (*p* < 0.05). These results demonstrate that the virion membrane potential is inherently modified following addition of variable concentrations of acetyl-CoA, a known regulator of cellular TCA cycle, and further highlighting the possible existence of a TCA cycle in *P. massiliensis*.

### Bioinformatics analyses reveal low similarities for 8 ORFs of *P. massiliensis* with the TCA cycle

Detailed bioinformatics analyses are included in Supplementary File 2. Using DELTA-BLAST analyses against the CDD [21], low amino acid sequence similarity with some enzymes involved in cellular TCA cycle were found. Before concluding that this similarity was not significant, we searched for other predicted *P. massiliensis* gene products with similarities to other enzymes of the TCA cycle. Low similarities were found for 8 *P. massiliensis* predicted gene products: citrate synthase (ORF577), aconitase (ORF1245), isocitrate/isopropylmalate dehydrogenase (ORF132 and ORF864), alpha-ketoglutarate decarboxylase (ORF762), (which converts alpha-ketoglutarate to succinic semialdehyde, which is an alternative pathway with the succinic semialdehyde dehydrogenase of the two steps of alpha-ketoglutarate dehydrogenase and succinate thiokinase), the succinate dehydrogenase (ORF181) and fumarase (ORF206). In addition, acetyl-CoA synthetase (ORF595), the immediate step upstream of the first step of the TCA cycle, was identified. Searching for domain similarity or structure of the predicted eight gene products with HHPRED, hmm search and Phyre2 was inconclusive. A BLASTp analysis of these 8 *P. massiliensis* ORFs revealed three of them to be conserved in some other pandoraviruses but not in all (Supplementary File 2).

One may suppose that if the *P. massiliensis* TCA genes are important for this virus, they should be conserved in other pandoraviruses too. However, it should also be noted that it is not always the case in the nature, as has been demonstrated in cyanobacteria. The TCA cycle of cyanobacteria is characterized by the absence of 2-oxoglutarate dehydrogenase, which is replaced by 2 alternative enzymes (alpha-ketoglutarate decarboxylase and succinic semialdehyde dehydrogenase). This may also be the case for *P. massiliensis*. Interestingly, not all cyanobacteria have these two alternative enzymes [52]. One can hypothesize an analogous scenario for pandoraviruses. For example, variant TCA cycles have been associated with low oxygen conditions for cyanobacteria, and reveal an essential adaptation and important plasticity to cope with readjustment to stressing environmental conditions. It also raises additional questions about a potential evolutionary advantage for the viruses harboring these genes whereas other could “survive” without them.

The search for predicted *P. massiliensis* proteins potentially involved in energy metabolism was unsuccessful for all enzymes except those in the TCA cycle, with low percentages of identity.

The TCA cycle is the central metabolic hub of cells. It is an exergonic catabolic energy acquisition pathway, which results in the oxidation of an acetyl group (derived from carbon compounds) to two molecules of carbon dioxide with the concomitant harvesting of high-energy electrons. Those electrons generate a proton gradient across the inner mitochondrial membrane through oxidative phosphorylation with the aim of producing ATP catalyzed by ATP synthase [53] The TCA cycle also provides, among other things, oxaloacetate for gluconeogenesis and metabolic intermediates for amino acid biosynthesis, nucleotide bases, cholesterol and porphyrins [53, 54]. Of note, not all eukaryotes have mitochondria [55, 56] and the TCA cycle can occur in anaerobic organisms utilizing fumarate, nitrate, or various other compounds as terminal electron acceptors instead of O_2_ [57, 58].

As *P. massiliensis* is neither a eubacterium nor a eukaryote, the role of a partial TCA cycle and the existence of a membrane potential in mature viral particles is currently enigmatic. In mitochondria and bacteria, the membrane potential allows cells to function as a battery and generate energy. In eukaryotic cells, mitochondrial membrane potential results in the production of ATP via the TCA cycle. For *P. massiliensis*, we could not detect the production of ATP in mature particles (data not shown). Currently, given the huge number of encoded hypothetical proteins and poor of knowledge about these recently discovered pandoraviruses, it is highly probable that these eight predicted *P. massiliensis* ORFs may be involved in alternative metabolic pathways. To date, only a few viral genes have been suggested to be involved in metabolic pathways such as in fermentation, sphingolipid biosynthesis and nitrogen metabolism [13]. *Ostreococcus tauri* viruses (Otv1-4) encode an ammonium transporter, which enables host growth rescue when cultured with ammonium as the sole nitrogen source [41]. TetV-1 encodes a mannitol metabolism enzyme, a saccharide degradation enzyme as well as other key fermentation genes [59]. In all these previous cases, the viral genes appear to be host-derived and are considered to be involved in viral manipulation of host metabolism. It has also been shown that some giant virus genomes, including pandoraviruses, contain cytochrome P450 genes which encode monooxygenase enzymes that are known to be essential in the metabolism of endogenous regulatory molecules and xenobiotics. However ancillary enzymatic redox partners encoded in theses viral genomes have not been identified to-date suggesting they may be recruited from host [12]. Recently, a deep analysis of 501 environmental metagenome-assembled genomes of NCLDV revealed a diversity of metabolic genes involved in nutrient uptake, light harvesting, nitrogen metabolism, glycolysis and the TCA cycle [60]. Interestingly, BLASTp analysis of the 8 *P. massiliensis* TCA ORFs against these metagenomes [60], and against those reported by Schulz et al. [61] did not show any significant matches. Moreover, tupanviruses possess a gene encoding a putative citrate synthase [14], the first enzyme in the TCA cycle, for which no homologs have been found in any other known virus. Phylogenetic analyses indicate an independent origin of this gene in tupanviruses, suggesting it may have been acquired by tupanviruses via horizontal gene transfer from sympatric bacteria.

Phylogenetically, it has been proposed that the origin of the TCA cycle may be the reductive TCA (rTCA, also called reverse TCA) cycle an endergonic anabolic pathway [62] used by many anaerobic, microaerobic but also by some aerobic bacteria to produce carbon compounds [62–70], and is known as one of the most ancient autotrophic metabolic pathways utilizing reductive acetyl-CoA [71]. The rTCA cycle shares the same enzymes of the TCA cycle in anaerobes except for the citrate cleaving enzymes (ATP citrate lyase, or its alternatives citryl-CoA synthetase and citryl-CoA lyase). One structural study suggested that an ancestral tetrameric citryl-CoA lyase, an enzyme from the reverse TCA cycle may have evolved towards the current ATP citrate synthase [72]. Congruently, a phylometabolic analysis showed the earlier occurrence of rTCA cycle than TCA cycle [73]. Inversely, other phylogenetic analyses of the citrate cleaving enzymes reveal that they have undergone major horizontal gene transfers, suggesting that the origin of life may not be autotrophic and thus that the rTCA cycle may not be the ancestor of the TCA cycle. The rTCA cycle may have evolved as a bacterial idiosyncratic pathway [71]. However, the evolutionary history of the TCA cycle is uncertain, although it has been suggested that prior to endosymbiotic events, this pathway only operated as isolated steps [74]. Consequently, the origin of its enzymes might be associated with lateral gene transfer or duplication events, suggesting other possible metabolic functionalities than those that are currently known [75]. A selection of 1408 representative hits from cellular organisms resulting from a BLASTp analysis of the COG0473 (isocitrate/isopropylmalate dehydrogenase that was aligned with the ORF132 of *P. massiliensis*) against the nr database, allowed building a phylogenetic tree (Supplementary Files 3, 4, 5). This reconstruction showed that the three orthologs found in pandoraviruses (ORF132 from *P. massiliensis*, ORF339 from *P. braziliensis*, and ORF902 from *P. neocaledonia*) were closely related and grouped apart from another cluster composed of *Thermoprotei archaeon* (RLG74176.1), *Methanobacterium aggregans* (WP209583414.1), *Methanothermobacter sp* (HHY00088.1) and *Methanobacteriaceae archaeon* (NYB28041.1). However, for the other 7 ORFS of *P. massiliensis*, the absence of any robust sequence identity with known proteins, prevented phylogenetic analyses and thus the determination of the evolutionary origin of these genes. This finding again raises the question of the role of such huge genomic information reservoirs harbored by pandoraviruses, in evolutionary processes (and convergent evolution) and their role(s) in lateral gene transfers. Our data argue that new gene candidates must be considered in the search for the origin of this cycle.

### The eight ORFs of *P. massiliensis* with similarity with the TCA cycle are transcribed during the replication cycle

Transcriptomic analysis of *P. massiliensis* during the viral replication cycle in *A. castellanii* has been previously reported [15]. Briefly, a time-course of transcriptomic analysis was carried out at 30 min, 2 h, 4 h, 6 h, and 8 h post-inoculation. The data revealed that 13% of viral transcripts were detected at H4 postinfection, more than 69% at H6 postinfection and 18% detected at H8 postinfection. The total number of the viral transcriptomic reads matched with 359 *P. massiliensis* ORFs (25% of the total gene content) [15].

**Fig. 4:**
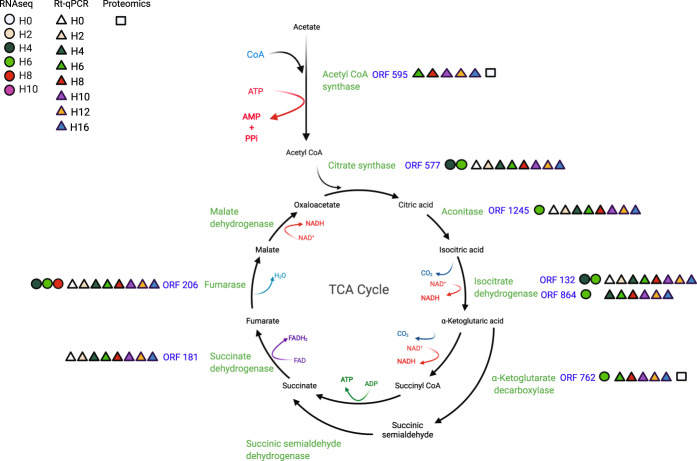
Recapitulative diagram of results obtained for each step of the TCA cycle. Schematic representation of the TCA cycle showing the predicted ORFs of *P*. *massiliensis* with similarities to TCA cycle enzymes, and a summary of the results provided by qRT-PCR, RNA sequencing and proteomics.

RNA sequencing revealed that 6 of the 8 predicted *P. massiliensis* ORFs were transcribed at different time points of the viral cycle, especially between H4 and H8 postinfection (Fig. 4). No transcripts were detected for ORF595 (putative acetyl-coenzyme A synthetase) or ORF181 (putative succinate dehydrogenase). qRT-PCR revealed that the 8 ORFs were transcribed at different time points of the viral cycle. For ORFs 595, 577, 1245, 132, 864, 762, and 206, the lowest Ct values were globally found between H8 and H16 postinfection. (Supplementary File 6).

Finally, transcriptomics analyses demonstrated that the predicted *P. massiliensis* TCA ORFs were all transcribed at the same time points, especially at the end of the developmental cycle of the virus (Fig. 4).

### Two of eight ORFs of *P. massiliensis* with similarity with the TCA cycle are expressed in mature virions

Proteomic analysis allowed us to identify 623 proteins with significant matches to known proteins in the amoeba/virus database. Among them, 96.8% (603 proteins) were derived from the host amoeba organism and only 3.2% (20 proteins) were identified as viral proteins. In a previous work, 162 protein sequences have been identified in mature virions [15]. Two *P. massiliensis* proteins of the eight predicted to be potentially involved in a partial TCA pathway were identified by this analysis in mature particles: ORF762 (putative α-ketoglutarate decarboxylase) and ORF595 (putative acetyl-coenzyme A synthetase) with an identity percentage of 100% (Fig. 4).

### Characterization of enzymatic functions

Genes encoding the predicted enzymes of interest were synthesized, cloned and transformed into competent *E. coli* for recombinant protein expression. Soluble proteins were obtained for ORFs 577 (citrate synthase), 1245 (aconitate hydratase), 132, 864 (isocitrate dehydrogenase), 787 and 1146 (α-ketoglutarate decarboxylases). However, no enzymatic activity could be observed for ORFs 577, 1245, 787 and 1146 (data not shown). We investigated the potential isocitrate dehydrogenase activity of ORFs 132 and 864. We determined a specific activity of 4 mU/mg for ORF132 (Supplementary Fig. 7), but no activity for ORF864. Kinetic assays were also performed to evaluate the catalytic parameters of ORF132. According to Michaelis–Menten equation fitting (*R*^*2*^ = 0.993), the following parameters were determined: kcat = 6.8 × 10^−4^ s^−1^, Km = 51.8 µM and kcat/Km = 13.12 s^−1^M^−1^ (Supplementary Fig. 8). The IDH activity assessed on purified mature virions was similar to those of the recombinant ORF132. In parallel, we performed the same analysis of human IDH from Sigma-Aldrich (St. Louis, MS, USA) and could determine a specific activity of 6.3 U/mg using the previously mentioned kit, as well as a kcat = 16.3 s^−1^, Km = 585.4 µM and kcat/Km = 2.78 × 104 s^–1^ M^−1^ with an *R*^*2*^ of 0.997. In the TCA cycle, isocitrate dehydrogenase converts the isocitrate to α-ketoglutarate in the presence of the NAD+ or NADP+ cofactor. In nature, isocitrate dehydrogenase catalyzes a catabolic reaction, during which NAD+ abstracts a hydride ion, a highly stereospecific enzymatic mechanism. Thus, the functionality of *P. massiliensis* isocitrate dehydrogenase cannot simply be the result of chance.

In cells, the IDH step of the TCA cycle is an irreversible reaction, with an overall estimated free energy of −8.4 kJ/mol [76]. It is regulated by substrate availability, product inhibition, and competitive feedback inhibition by ATP [77]. Isocitrate binds within the enzyme active site, involving 8 key amino acids. The metal ion Mg^2+^ or Mn^2+^ binds to three conserved Arg residues through hydrogen bond networks. The cofactor NAD^+^ or NADP^+^ binds within four regions with similar properties among the IDH enzymes, located around amino acids [250–260], [280–290], [300–330], and [365–380] [78]. ORF132 is 146 amino acids long, while the known IDH from *E.coli* is 417 long (QJZ24410.1). IDH is typically multimeric [79] suggesting that the pandoraviral enzyme may also form multimers. If the pandoraviral IDH is an ancestral form of the current eukaryotic IDH, then it is not surprising that the Km and Kcat of ORF132 are low. Evolutionary, enzymes involved in the TCA cycle have evolved towards optimal performance with higher Km and Kcat values.

## Conclusion and perspectives

Experimental evidence of a virus encoding enzymes of a partial TCA cycle opens a new horizon in giant virus research. For each experiment, critical negative controls were carried out to avoid false positive results. Membrane potential and in particular, membrane depolarization, have been previously described in *Chlorella* viruses, and have been linked to K+ protein channels [80, 81]. Therein, the fusion of the virus internal membrane with the host plasma membrane results in the membrane depolarization that occurs early in *Chlorella* virus-host interactions. This phenomenon, lowering the energy barrier, allows the viral DNA release into the host. However, it appears to differ from those observed in pandoraviruses. Indeed, pandoraviruses seem to harbor predicted genes with low similarity with all enzymes of the cellular TCA cycle and most importantly, a functional isocitrate dehydrogenase, a key enzyme of this cycle. Moreover, the membrane potential of *P. massiliensis* virions is affected by acetyl-CoA, a well-known regulator of the TCA cycle. One may assume that a complete TCA cycle can occur if all the enzymatic components are present in a system. Herein, our findings suggest the possible existence of an autonomous, at least partial, TCA pathway potentially involved in energy production, or in another, unknown metabolic pathway, in a virus. As the TCA cycle plays a key role in anabolism and catabolism of many biomolecules, it also can also be hypothesized that these viral genes may be involved in redirecting amoeba carbon metabolism.

It is known that viruses are major drivers in nutrient and energy recycling within natural environments and in the ecology and evolution of their cellular hosts [82–85]. In the setting of host-virus interactions, the ability of the viruses to generate energy would increase their own fitness. Indeed, one can imagine that the delivery of reducing power (NADH or NADPH) through the putative virus-encoded TCA enzymes into the host cell upon infection can provide a significant increase of direct usable energy to sustain the initial step of viral replication without affecting host cell needs. This can be comparable to cyanoviruses, which carry horizontally transferred auxiliary metabolic genes that presumably maintain functional photosynthetic machinery during infection and supply energy to the bacterial host, as transcription of host genes drops during infection [38, 86–88]. The prevalence of such auxiliary metabolic genes in the environment [45, 89, 90] raises important questions regarding their contribution in the biosphere [45]. In the light of this study, we have to rethink and take into account viral input(s) in energy storage and flux pathways within the microbial food web. Future experiments are clearly needed for understanding fully the existence of a TCA cycle in such viruses and are underway in our laboratories. It should be noted the limit of the standard comparative genomics tool in identifying novel, or small and very phylogenetically divergent sequences. Experiments investigating the crystal structures of the viral enzymes, especially those of IDH, will be of particular interest for advancing comprehension of this mysterious Pandoravirus. Moreover, the use of yeast and bacterial complementation experiments to confirm the predicted enzymatic functions seems to be the next logical steps. The database of newly discovered giant viruses is constantly growing and constitutes an important resource for the search for similar predicted enzymatic functions potentially involved in metabolic pathways.

In conclusion, *P. massiliensis* undermines the last known historical viral hallmark, the lack of the Lipman system. Thus, our findings presented herein raise critical questions concerning whether pandoraviruses can still be accurately classified biologically as viruses and renew deep-rooted arguments regarding the living nature of viruses in general.

## Supplementary information

Supplementary file 1

Supplementary file 2

Supplementary file 3

Supplementary file 4

Supplementary file 5

Supplementary file 6

Supplementary figure 1

Supplementary figure 2

Supplementary figure 3

Supplementary figure 4

Supplementary figure 5

Supplementary figure 6

Supplementary figure 7

Supplementary figure 8

Legends for supplementary files/figs
