# Correction to: Incomplete tricarboxylic acid cycle and proton gradient in *Pandoravirus massiliensis*: is it still a virus?

**DOI:** 10.1093/ismejo/wraf006

**Published:** 2025-11-18

This is a second correction to: Sarah Aherfi, Djamal Brahim Belhaouari, Lucile Pinault, Jean-Pierre Baudoin, Philippe Decloquement, Jonatas Abrahao, Philippe Colson, Anthony Levasseur, David C Lamb, Eric Chabriere, Didier Raoult, Bernard La Scola, Incomplete tricarboxylic acid cycle and proton gradient in *Pandoravirus massiliensis*: is it still a virus? *The ISME Journal*, Volume 16, Issue 3, March 2022, Pages 695–704, https://doi.org/10.1038/s41396-021-01117-3

In June 2024, a correction was published regarding Figure 3, panel A2[1]. In November 2024, an additional concern was raised regarding Figure 3, panels C2 and D2. Upon review, the authors confirmed panel C2 (representing the 200 µM condition) had mistakenly been duplicated in panel D2 (representing the 300 µM condition).

This duplication occurred during figure assembly and does not represent the actual experimental condition.

The quantitative measurements for all data at each specific condition were not impacted by this mistake, and the original results, discussion, and conclusions remain unchanged.

The authors have replaced panel D2 in Figure 3 below.

**Figure:**
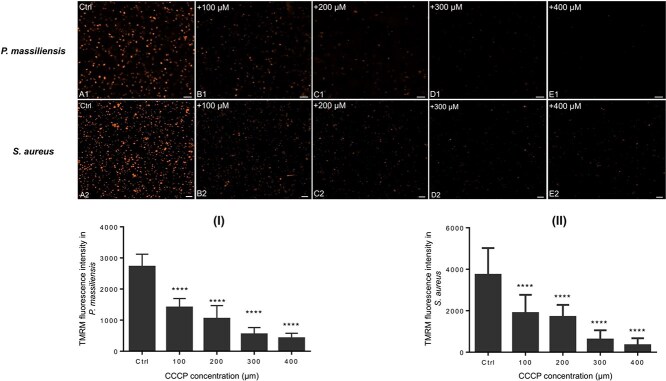


The authors sincerely apologise unreservedly for this second error.

The error is outlined only in this notice to preserve the version of record.

## Footnotes

^1^
Correction to: Incomplete tricarboxylic acid cycle and proton gradient in *Pandoravirus massiliensis*: is it still a virus? *The ISME Journal*, Volume 18, Issue 1, January 2024, wrae095, https://doi.org/10.1093/ismejo/wrae095

