# Supplementary material for: Incomplete tricarboxylic acid cycle and proton gradient in Pandoravirus massiliensis: is it still a virus?
Source: ISME J. 2021 Sep 23;16(3):695–704. doi: 10.1038/s41396-021-01117-3 (PMC8857278; doi:10.1038/s41396-021-01117-3)
Supplement: Supplementary file 1 [file 41396_2021_1117_MOESM1_ESM.docx]

**Supplementary file 1. qRT-PCR primers used in the present study of the predicted ORFs of *P. massiliensis.***

| **Predicted ORF** | **Predicted function** | **Sequences of the primers (5’-3’)** |
| --- | --- | --- |
| **ORF595** | Acetyl Co A Synthetase | F: CCCACCAAGCAATCTCTGTC  R: TACTGTGTGTGTGGGTAGGC |
| **ORF577** | Citrate synthase | F: TCTGGATGGCGTACGGAG  R: CGACTTTTCCTCGCCATCTG |
| **ORF1245** | Aconitate hydratase | F: TAGGATAGGGCGTCGGATTC  R: GAACAAGAAGGCACCAAGGG |
| **ORF132** | Isocitrate dehydrogenase | F: ATCCTGATCCATCCATGCGT  R: CGTCCCAGCACAAGAGTTTT |
| **ORF864** | Isocitrate dehydrogenase | F: TGTTGTCGGCACTTTCCAAG  R: CTTTTCCGTGAGCAGGTGAG |
| **ORF762** | α-ketoglutarate decarboxylase | F: TGTCTGTTTCTTGCCGAGTC  R: TTTCTTGGGCGCTTTCAGAG |
| **ORF181** | Succinate dehydrogenase | F: CAGTGGCCGATATTGTGCAA  R: GTCGATGGCAGCTACAAGAC |
| **ORF206** | Fumarase | F: TTGGGAGGAGTTGGTCTGTG  R: CGCTTGAGTTTGTCCGTGTT |
