# Supplementary material for: Incomplete tricarboxylic acid cycle and proton gradient in Pandoravirus massiliensis: is it still a virus?
Source: ISME J. 2021 Sep 23;16(3):695–704. doi: 10.1038/s41396-021-01117-3 (PMC8857278; doi:10.1038/s41396-021-01117-3)
Supplement: Supplementary file 2 [file 41396_2021_1117_MOESM2_ESM.docx]

**Supplementary file 2.**

Results of the bioinformatics analyses.

Using DELTA-BLAST analyses against the Conserved Domain Database (CDD), low sequence similarity with enzymes involved in the TCA were found. Before concluding that this similarity was not significant, we searched for other predicted *P. massiliensis* gene products with similarities to other enzymes of the TCA cycle (i.e., citrate synthase, aconitase, α-ketoglutarate dehydrogenase, succinyl CoA synthetase, succinate dehydrogenase, fumarase). Low similarities were found for 7 *P. massiliensis* predicted gene products with 6 enzymes of the TCA cycle. The product of ORF577 exhibited 33% identity to the conserved domain PRK05614 of citrate synthase (bitscore 58). A similarity of the ORF1245 gene product was found for domain pfam05681 of aconitase. The *P. massiliensis* ORF132 gene product harbored similarity to isocitrate/isopropyl malate dehydrogenase (COG0473) with a bitscore of 67 and 30% identity. Using a HHPRED with the TIGR PFAM database also revealed a low similarity of ORF132 with TIGR00169, an NAD or NADP dehydrogenase, including dimeric forms of IDH. In addition to the similarity found for ORF132, ORF864 harbored 50% identity to another domain of IDH (pfam03971) (bitscore: 54). No similarity was found for α-ketoglutarate deshydrogenase or succinate thiokinase. However, a low similarity was found for the *P. massiliensis* predicted ORF762 gene product (bitscore: 55; identity: 41%) with alpha-ketoglutarate decarboxylase. The ORF181 gene product was approximately 30% identical to domain PRK09078 of the succinate dehydrogenase (bitscore: 77). Finally, domain PRK06246 of fumarase showed 30% identity to the predicted ORF206 gene product (bitscore: 58). The search for a similarity of structure of these 7 ORFs with Phyre2 was inconclusive. No similarity was found for malate dehydrogenase. Of note, a hit with acetyl-CoA synthetase, the immediate step upstream of the first step of the TCA cycle (synthesis of citrate starting from acetyl-CoA) was found for the ORF595 gene product (bitscore: 58; identity: 24%).

BLASTp analyses of these 8 *P. massiliensis* ORFs putatively involved in the TCA cycle against the nr database revealed the predicted enzymatic function for only the gene product of ORF595, with only one hit annotated as acetyl CoA synthetase of *Phalacrocorax carbo*, with 33% identity. An ortholog in other pandoraviruses was also found for the ORF132, ORF181 and ORF206 of *P. massiliensis*, with significant bitscores and identity percentages.

All the results are summarized in the table below.

BLASTp analyses of these 8 *P. massiliensis* ORFs putatively involved in the TCA cycle against other pandoraviruses

| Orf number in *Pandoravirus massiliensis* | Predicted function | Hit | Orf number in the hit | e-value | bitscore | % identity |
| --- | --- | --- | --- | --- | --- | --- |
| ORF 132 | ISOCITRATE DEHYDROGENASE | *P. braziliensis* | MZ384235 (orf339) | 5.81E-47 | 146 | 61.1 |
| ORF 132 | ISOCITRATE DEHYDROGENASE | *P. neocaledonia* | YP_009482512.1_902 | 3.03E-45 | 142 | 60.1 |
| ORF181 | SUCCINATE DEHYDROGENASE | *P. macleodensis* | YP_009481470.1_786 | 1.52E-152 | 428 | 74.4 |
| ORF 181 | SUCCINATE DEHYDROGENASE | *P. neocaledonia* | YP_009482477.1_867 | 1.25E-144 | 408 | 70.6 |
| ORF 181 | SUCCINATE DEHYDROGENASE | *P. braziliensis* | MZ384236 (orf243) | 2.4E-142 | 402 | 70.5 |
| ORF181 | SUCCINATE DEHYDROGENASE | *P. salinus* | YP_008438591.1 | 1.14E-64 | 204 | 44.9 |
| ORF 181 | SUCCINATE DEHYDROGENASE | *P. celtis* | QBZ81659.1 | 3.95E-61 | 196 | 46.3 |
| ORF181 | SUCCINATE DEHYDROGENASE | *P. quercus* | YP_009483748.1 | 2.09E-60 | 194 | 43.8 |
| ORF181 | SUCCINATE DEHYDROGENASE | *P. inopinatum* | YP_009119137.1 | 1.61E-58 | 189 | 43.5 |
| ORF181 | SUCCINATE DEHYDROGENASE | *P. pampulha* | MZ384237 (orf2054) | 2.31E-58 | 189 | 48.6 |
| ORF181 | SUCCINATE DEHYDROGENASE | *P. japonicus* | BCU02985.1 | 8.92E-58 | 187 | 48.3 |
| ORF181 | SUCCINATE DEHYDROGENASE | *P. dulcis* | YP_008319862.1 | 5.29E-56 | 186 | 46.1 |
| ORF181 | SUCCINATE DEHYDROGENASE | *P. inopinatum* | YP_009118977.1 | 4.71E-08 | 51 | 31.6 |
| ORF181 | SUCCINATE DEHYDROGENASE | *P. dulcis* | YP_008319118.1 | 2.12E-07 | 49 | 35.2 |
| ORF 206 | FUMARASE | *P. neocaledonia* | YP_009482461.1 | 3.74E-81 | 239 | 71.9 |
| ORF 206 | FUMARASE | *P. macleodensis* | YP_009481453.1 | 5.42E-80 | 234 | 70.1 |
| ORF 206 | FUMARASE | *P. dulcis* | YP_008319938.1 | 1.38E-58 | 179 | 56.1 |
| ORF 206 | FUMARASE | *P. japonicus* | BCU03061.1 | 1.11E-57 | 177 | 55.1 |
| ORF 206 | FUMARASE | *P. pampulha* | MZ384238 (orf2207) | 1.29E-57 | 177 | 55.1 |
| ORF 206 | FUMARASE | *P. salinus* | YP_008438707.1 | 1.45E-55 | 172 | 52.8 |
| ORF 206 | FUMARASE | *P. quercus* | YP_009483811.1 | 6.61E-54 | 167 | 52.8 |
| ORF 206 | FUMARASE | *P. celtis* | QBZ81717.1 | 6.61E-54 | 167 | 52.8 |

Stringent DELTA-BLAST analyses for other pandoraviruses (e-value ≤ 1e-3 and identity ≥ 30% as thresholds) showed that 12 predicted translated ORFs had a hit against a domain of an enzyme involved in the TCA cycle, which was confirmed by BLASTp analysis against the nr database. These 12 ORFs putatively encode an acetyl-coenzyme A synthetase, a citrate synthase, an aconitase, a NADP-dependent IDH, a succinate dehydrogenase, and a malate dehydrogenase (See the table below). Moreover, DELTA-BLAST analysis revealed that a single translated ORF from *P. neocaledonia* (YP_009482013.1) harbored similarity to NADH dehydrogenase, an enzyme involved in the respiratory chain. This result was confirmed by BLASTp analysis against the nr database (bitscore: 45; identity: 31% with PKL55719.1, NADH:ubiquinone oxidoreductase from Methanomicrobiales archaeon HGW-Methanomicrobiales-6).

BLASTp analysis against the COG database provided a hit for COG0277 (FAD/FMN-containing dehydrogenase) in all but two (*P. celtis* and *P. macleodensis*) pandoraviruses, with e-values ranging from 9.5e-68 and 9.1e-62 and identity percentages between 30.6 and 33.5% for alignment lengths ranging from 514 to 532 amino acids.

A hit was also found in *P. salinus*, *P. dulcis*, *P. inopinatum* and *P. pampulha* for COG1254 (acylphosphatase), with e-values ranging from 2.1e-15 to 2.57e-12 and identity percentages from 23.5 to 28.2% for alignments ranging from 156 to 239 amino acids in length.

**Results of the DELTA-Blast analyses carried out on the pandoraviruses**

| **Virus** | **Protein** | **Genbank accesion num. of the hit** | **Predicted function** | **e-value** | **bitscore** | **% identity** |
| --- | --- | --- | --- | --- | --- | --- |
| ***P.inopinatum*** | YP_009119080.1 | GBF28309.1 | Acetyl-coenzyme A synthetase | 0.003 | 35.4 | 42 |
| ***P. pampulha*** | MZ384239 (Orf 1636) | RPB15391.1 | Citrate synthase | 1,56e-04 | 39.3 | 29 |
| ***P. celtis*** | QBZ81646.1 | WP_007415625.1 | Citrate synthase | 6,35e-04 | 35.4 | 32 |
| ***P. braziliensis*** | MZ384233 (Orf 702) | WP_038537937.1 | Aconitate hydratase AcnA | 5,53e-05 | 38.9 | 36 |
| ***P. neocaledonia*** | YP_009481720.1 | WP_016709093.1 | Bifunctional aconitate hydratase 2/2-methylisocitrate dehydratase | 0.002 | 34.3 | 36 |
| ***P. dulcis*** | YP_008318963.1 | WP_085890007.1 | NADP-dependent isocitrate dehydrogenase | 0.009 | 28.5 | 48 |
| ***P. dulcis*** | YP_008320016.2 | HAU16652.1 | Succinate dehydrogenase flavoprotein subunit | 2,76e-04 | 37.7 | 36 |
| ***P. braziliensis*** | MZ384234 (Orf 13) | WP_010797804.1 | Succinate dehydrogenase | 4,69e-06 | 38.5 | 58 |
| ***P. salinus*** | YP_008436514.1 | KAB2653497.1 | Fumarate reductase/succinate dehydrogenase flavoprotein subunit | 7,57e-04 | 32.3 | 48 |
| ***P. salinus*** | YP_008437242.1 | WP_162476042.1\| | Succinate dehydrogenase iron-sulfur subunit | 0.001 | 33.9 | 34 |
| ***P. dulcis*** | YP_008319894.1 | WP_085081987.1 | Fumarate reductase/succinate dehydrogenase flavoprotein subunit | 0.002 | 30.4 | 43 |
| ***P. salinus*** | YP_008438520.1 | CDJ81749.1 | Lactate malate dehydrogenase domain containing protein | 6,31e-05 | 37 | 32 |
