# Supplementary figures and images for: Incomplete tricarboxylic acid cycle and proton gradient in Pandoravirus massiliensis: is it still a virus?
Source: ISME J. 2021 Sep 23;16(3):695–704. doi: 10.1038/s41396-021-01117-3 (PMC8857278; doi:10.1038/s41396-021-01117-3)

Tree scale: 10

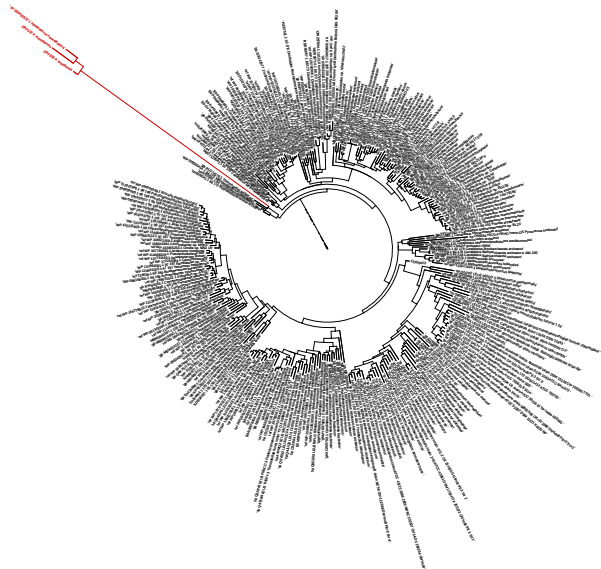

Supplement: Supplementary file 5 [file 41396_2021_1117_MOESM5_ESM.pdf]

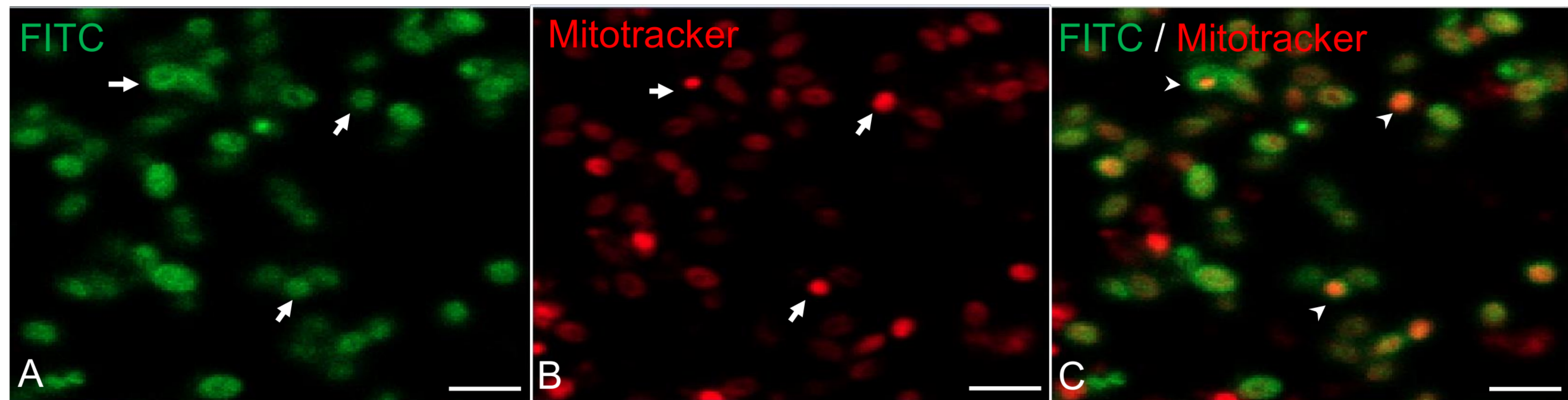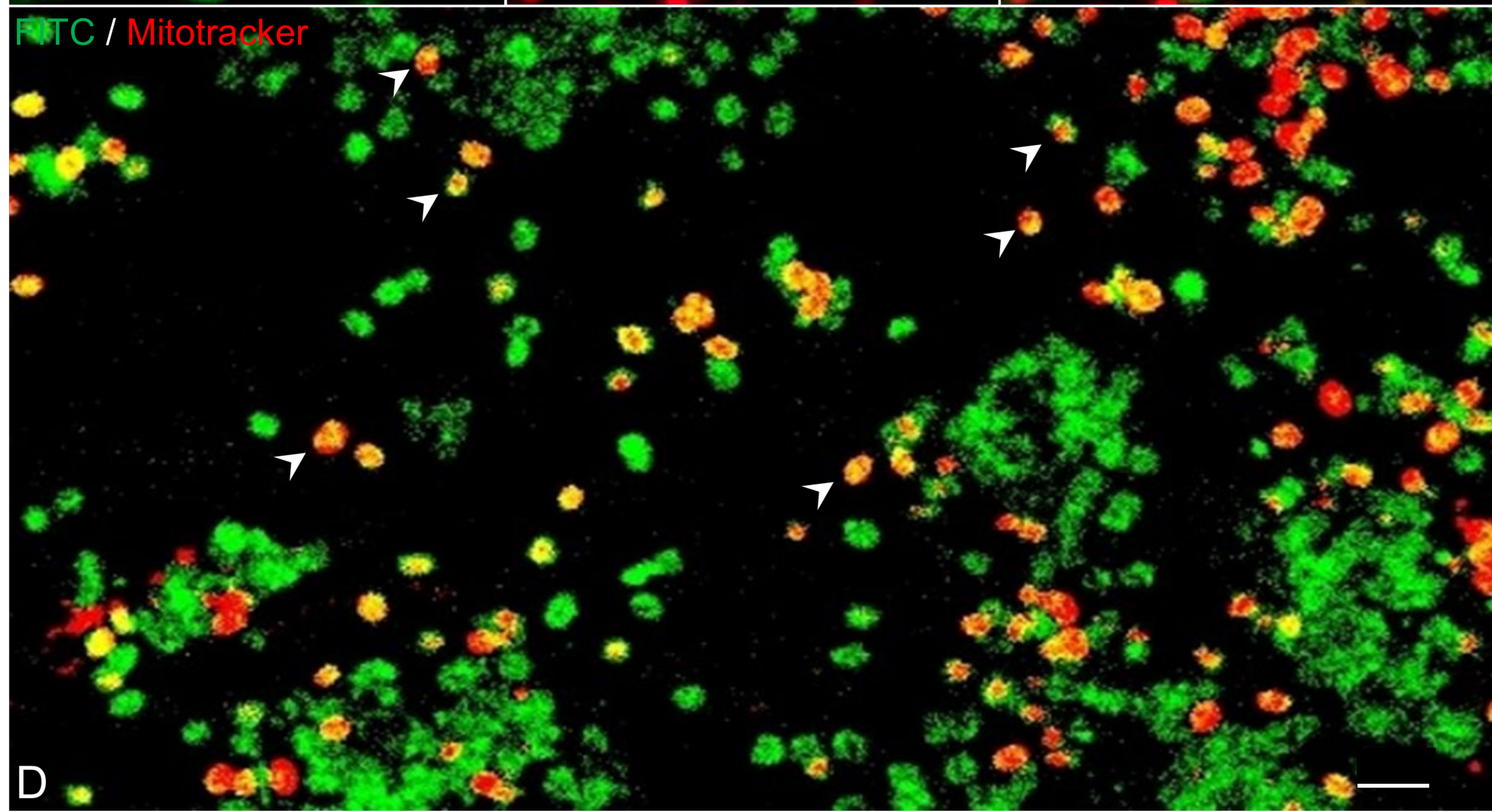

Supplement: Supplementary file 7 — Supplementary figure 1 [file 41396_2021_1117_MOESM7_ESM.pdf]

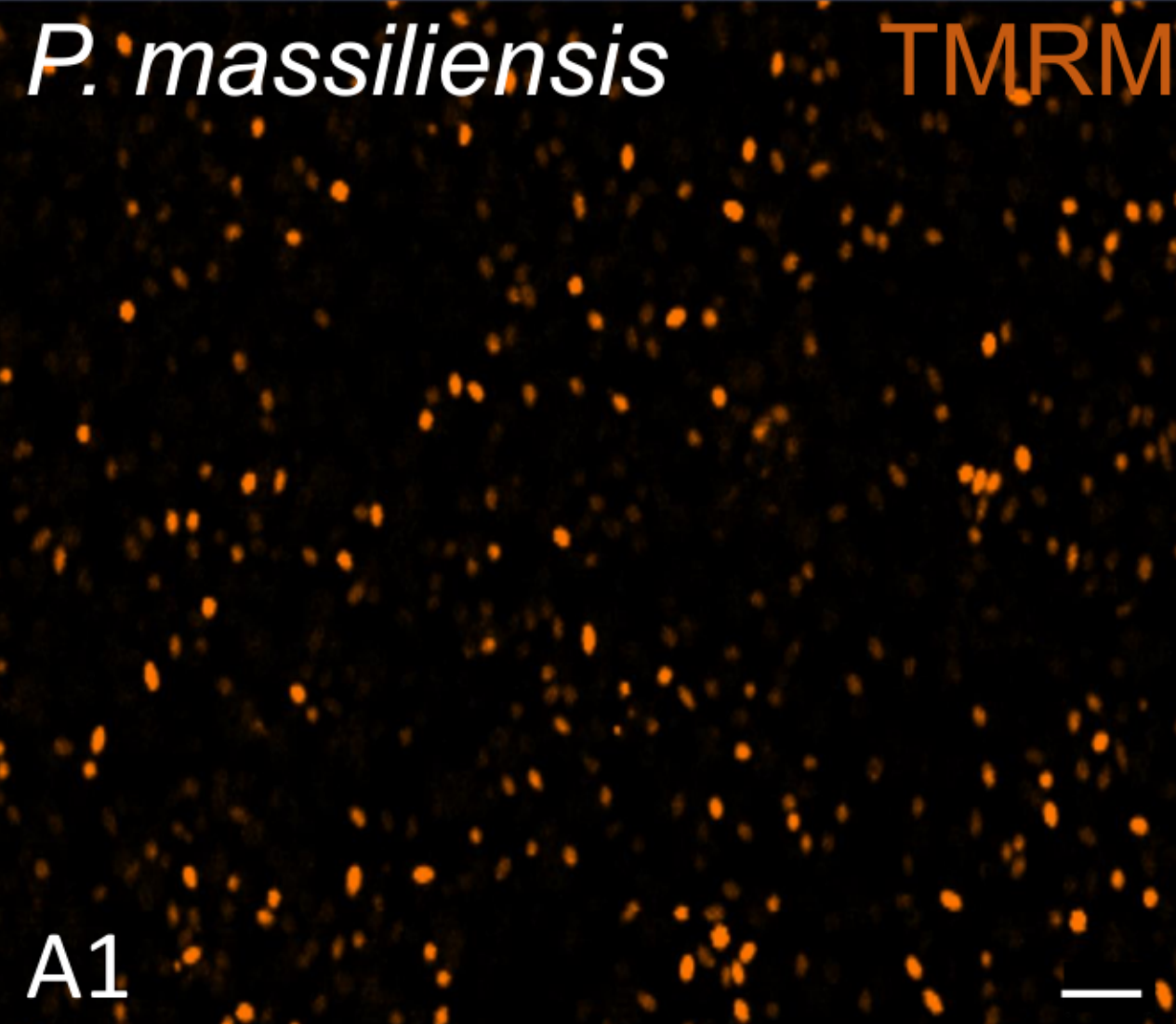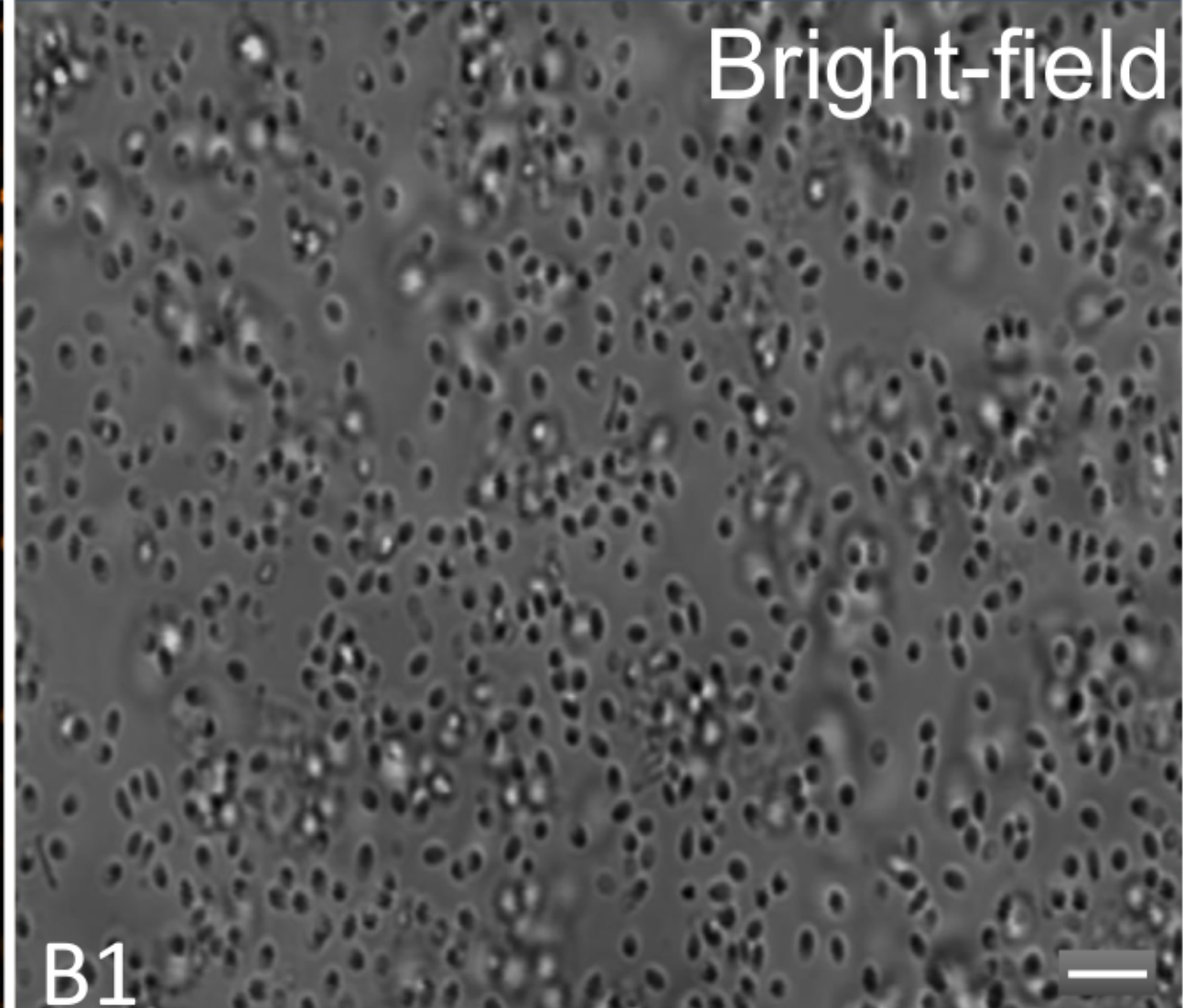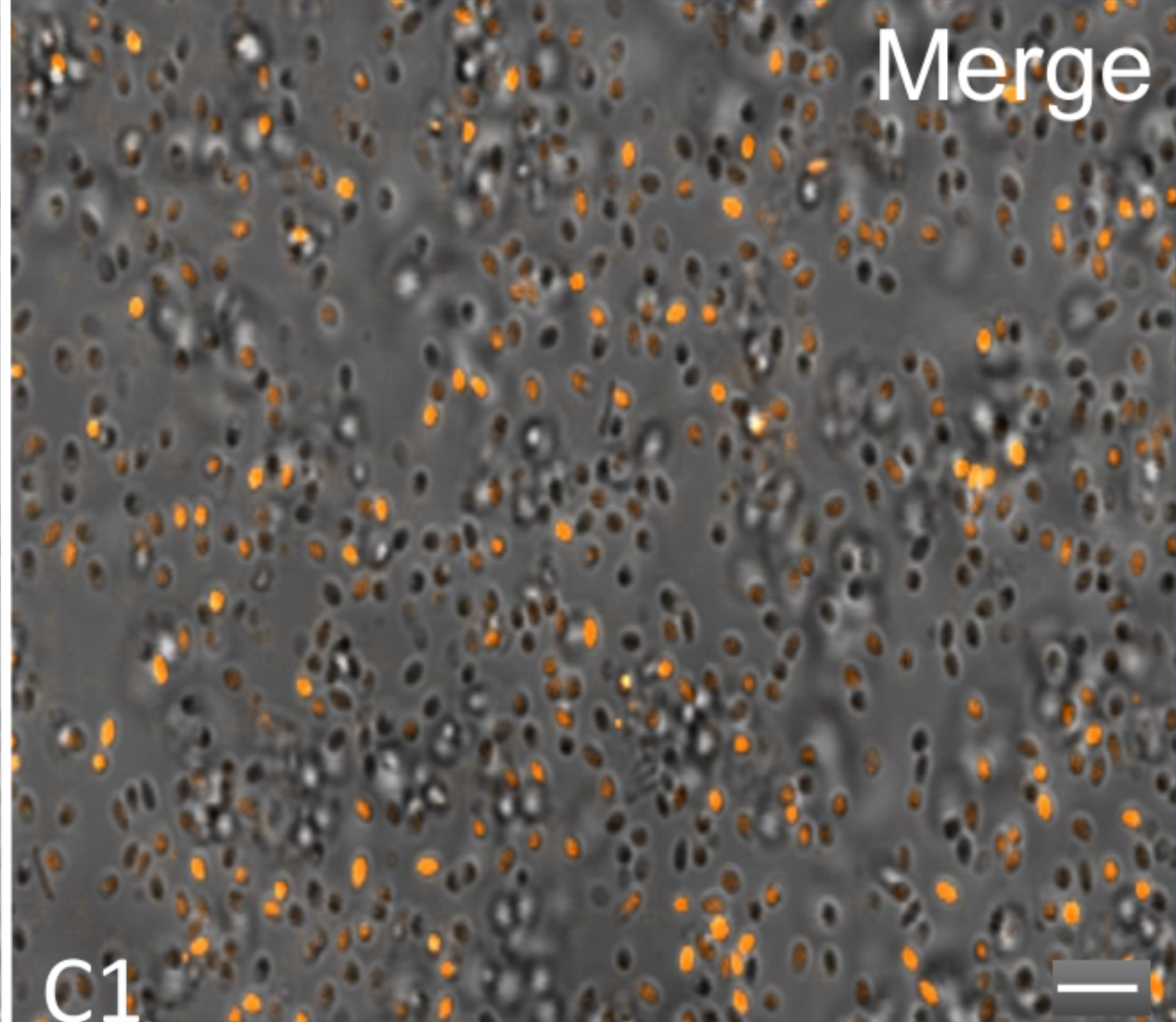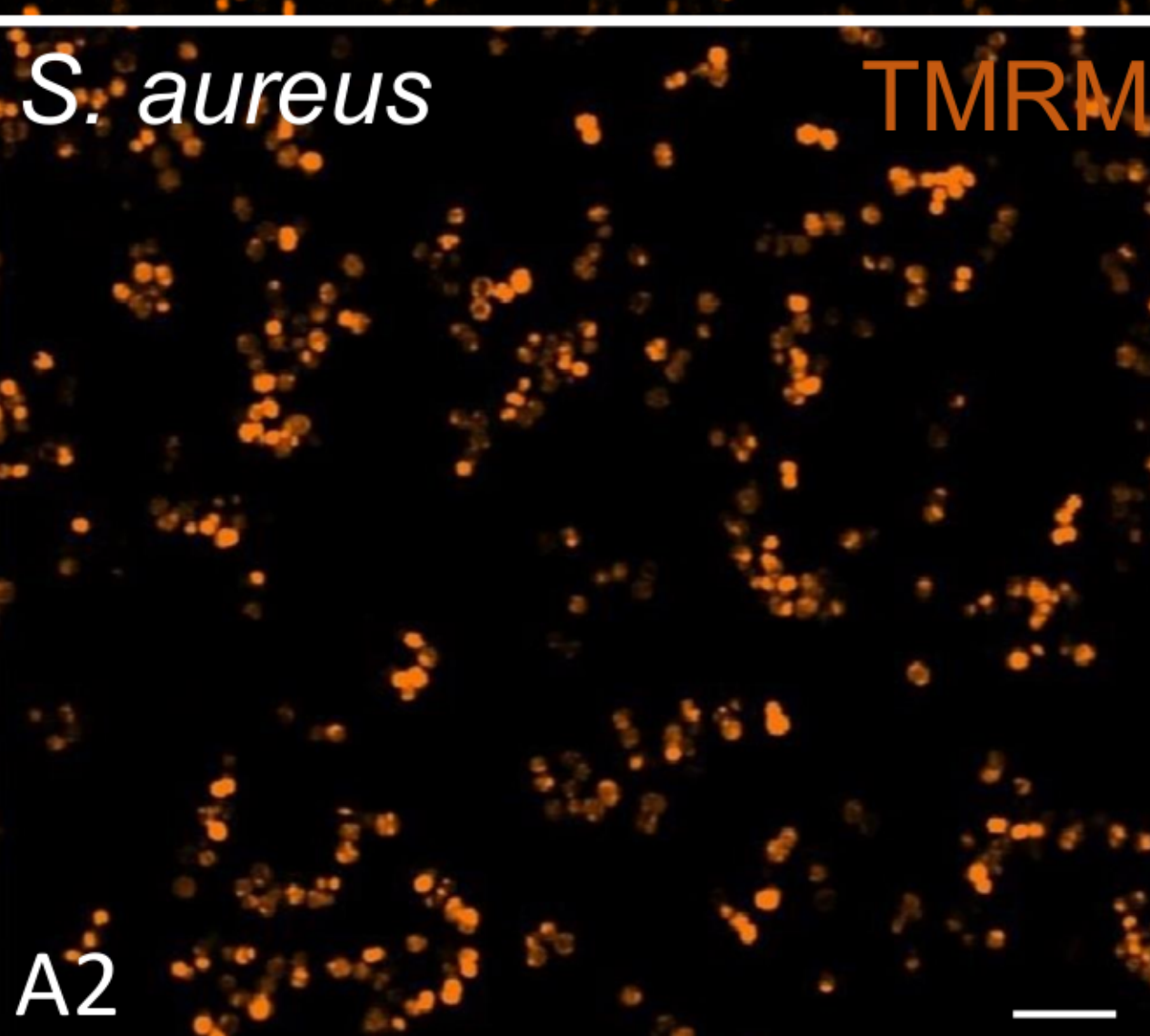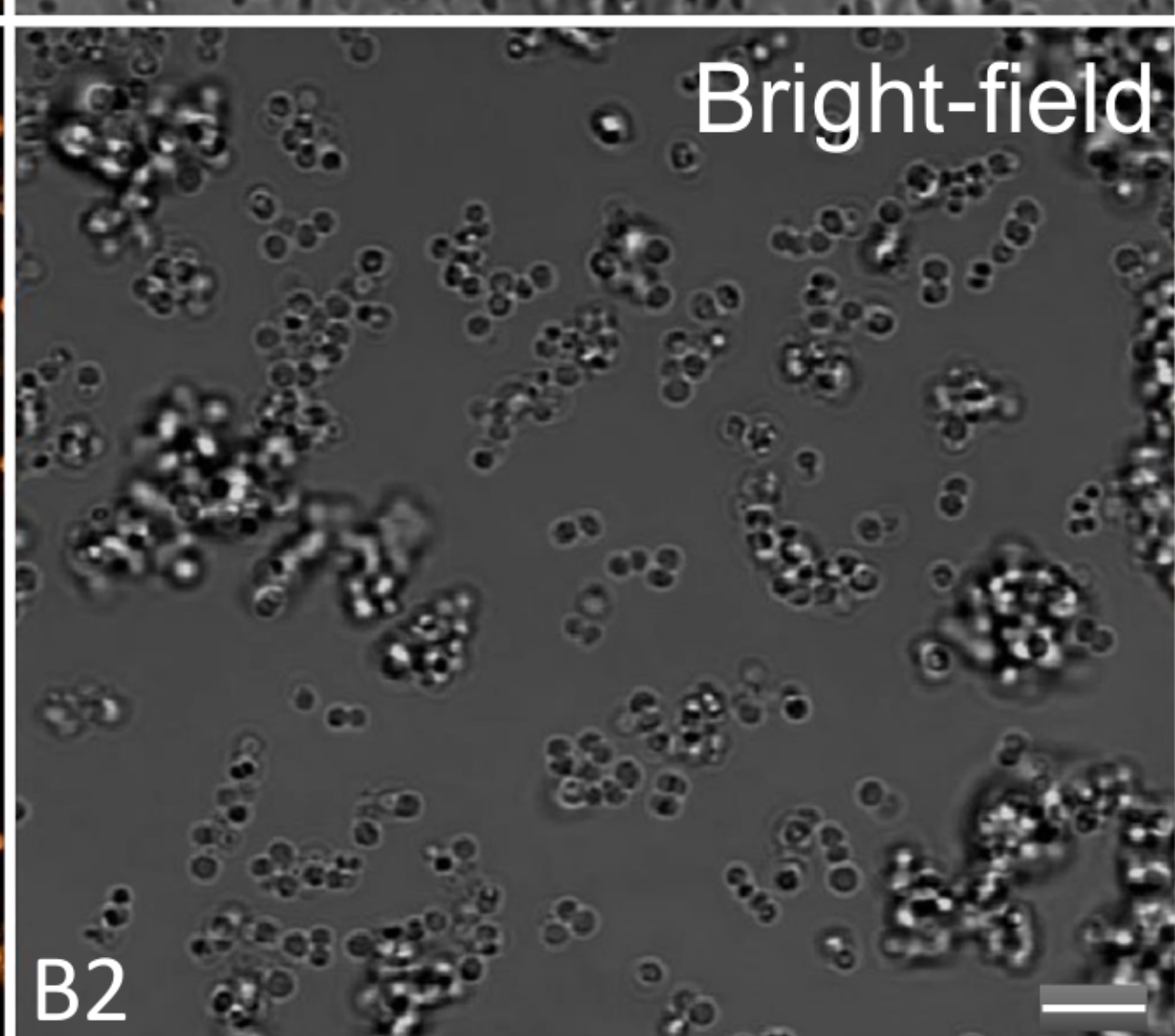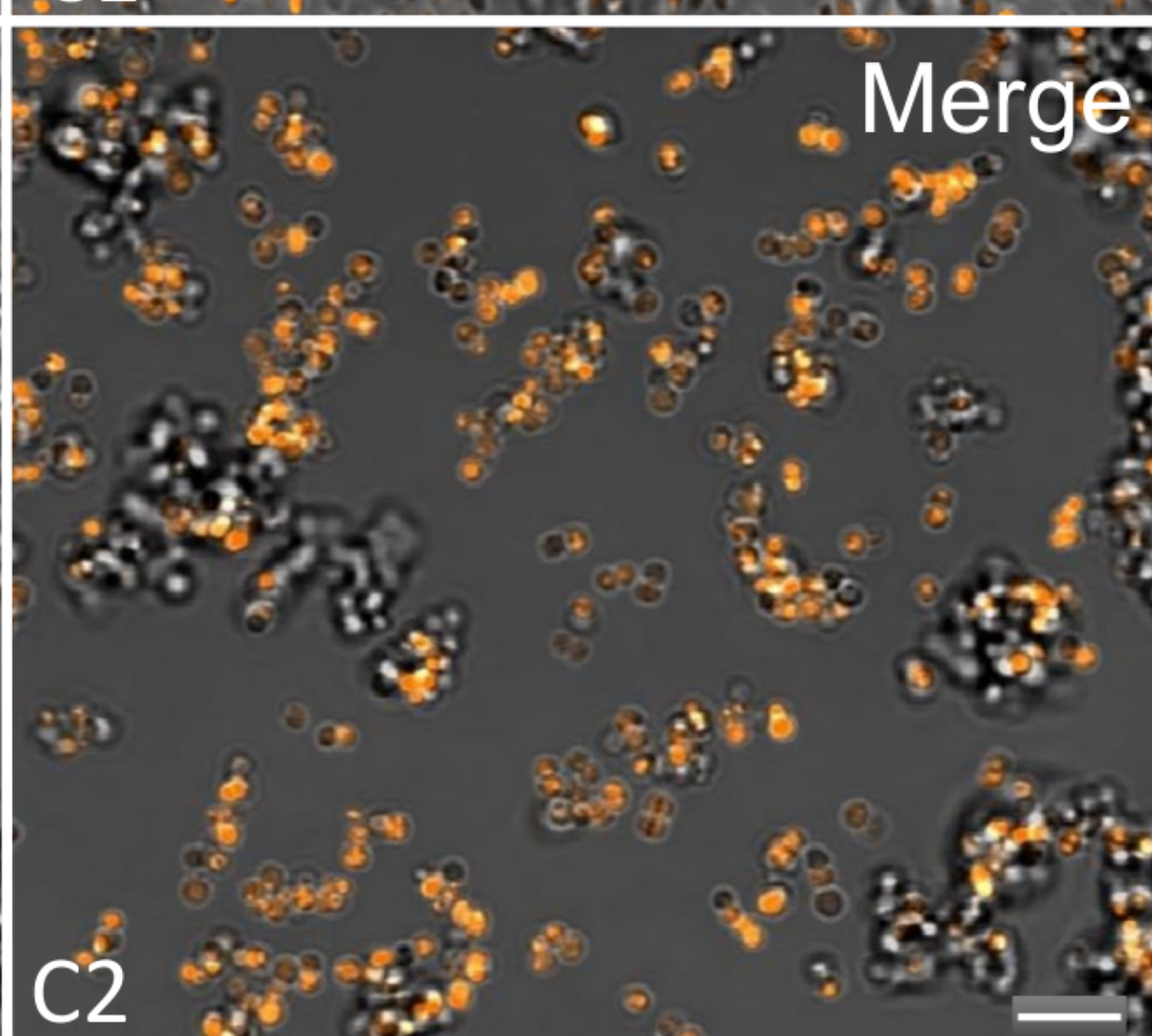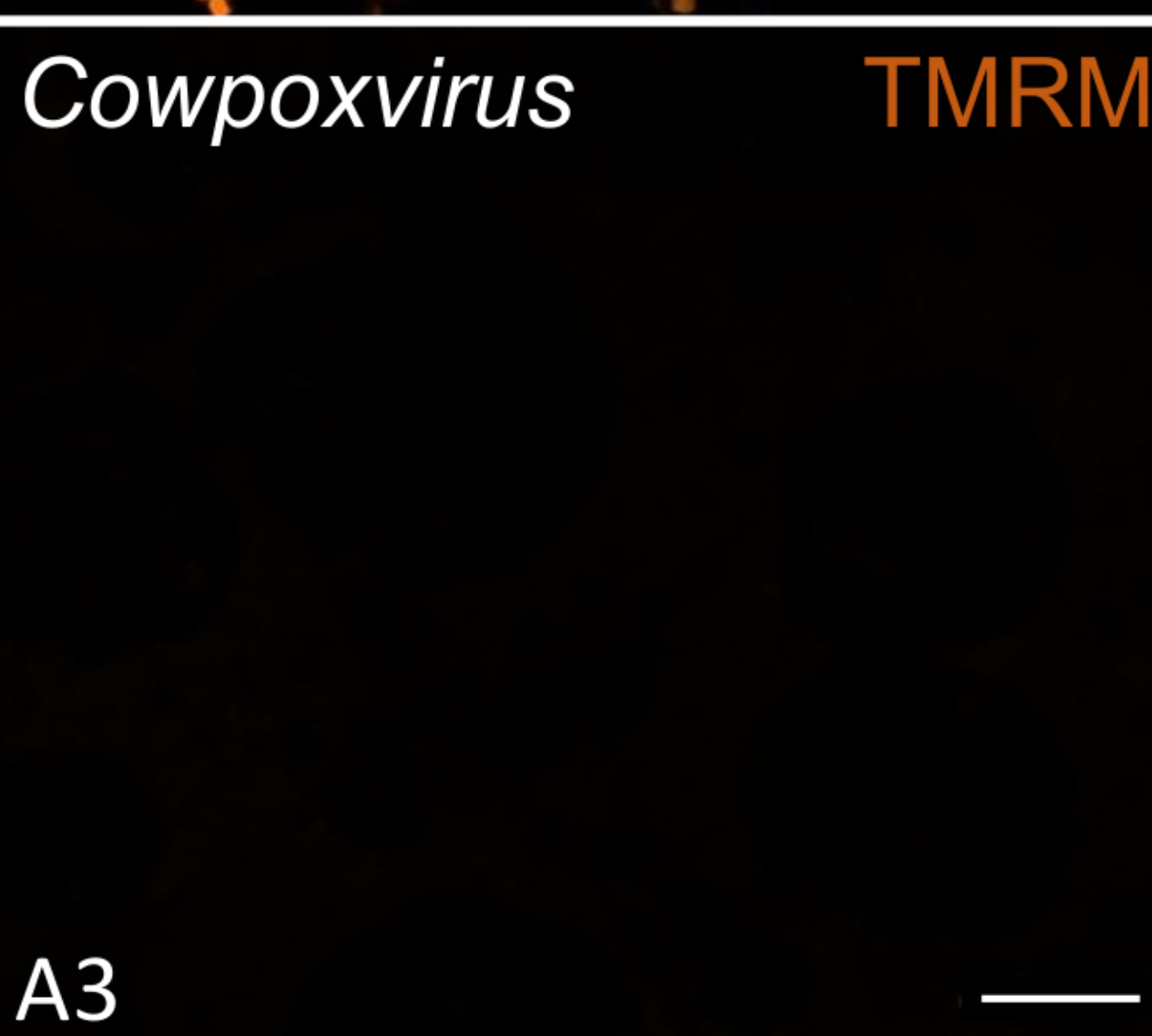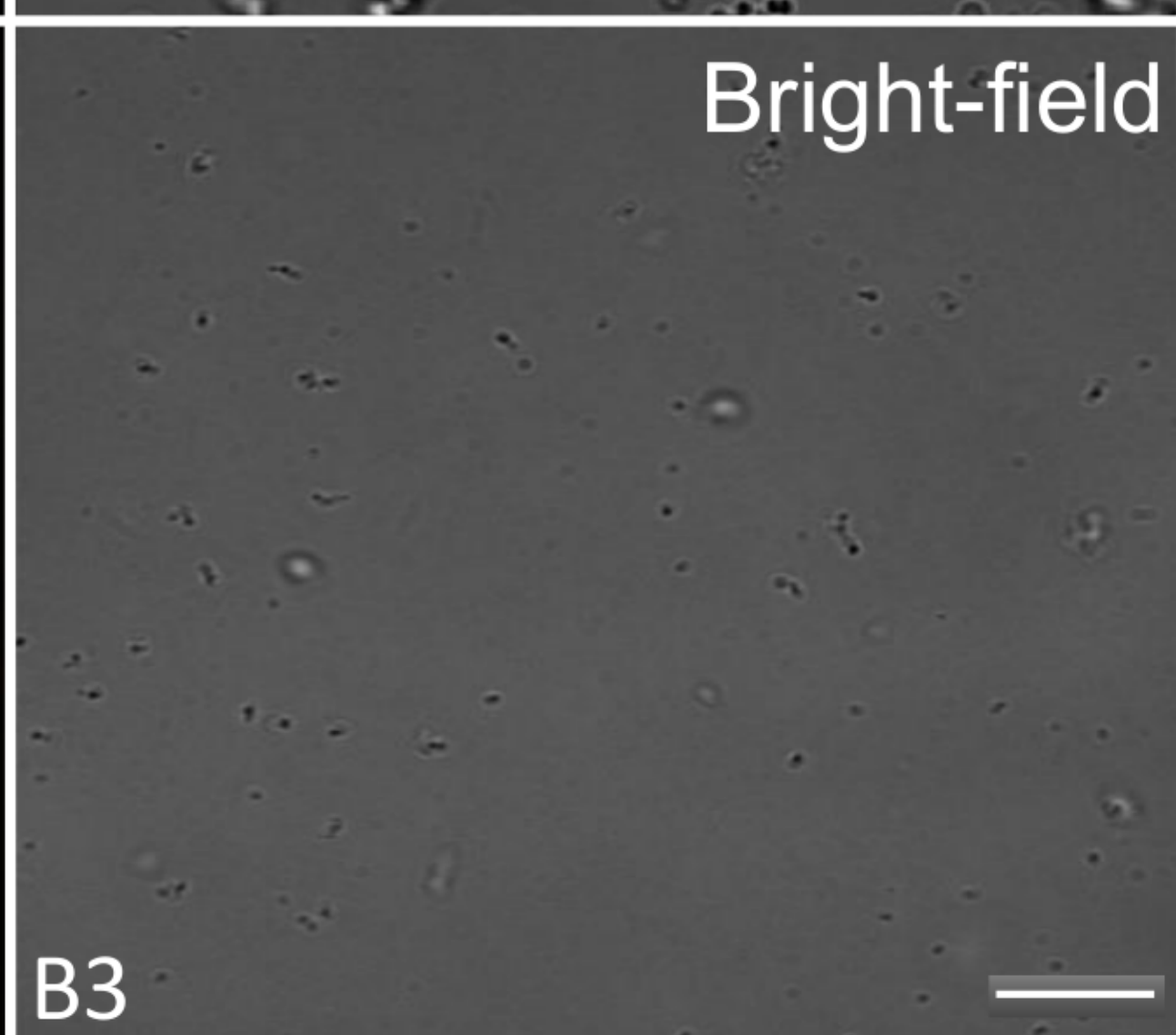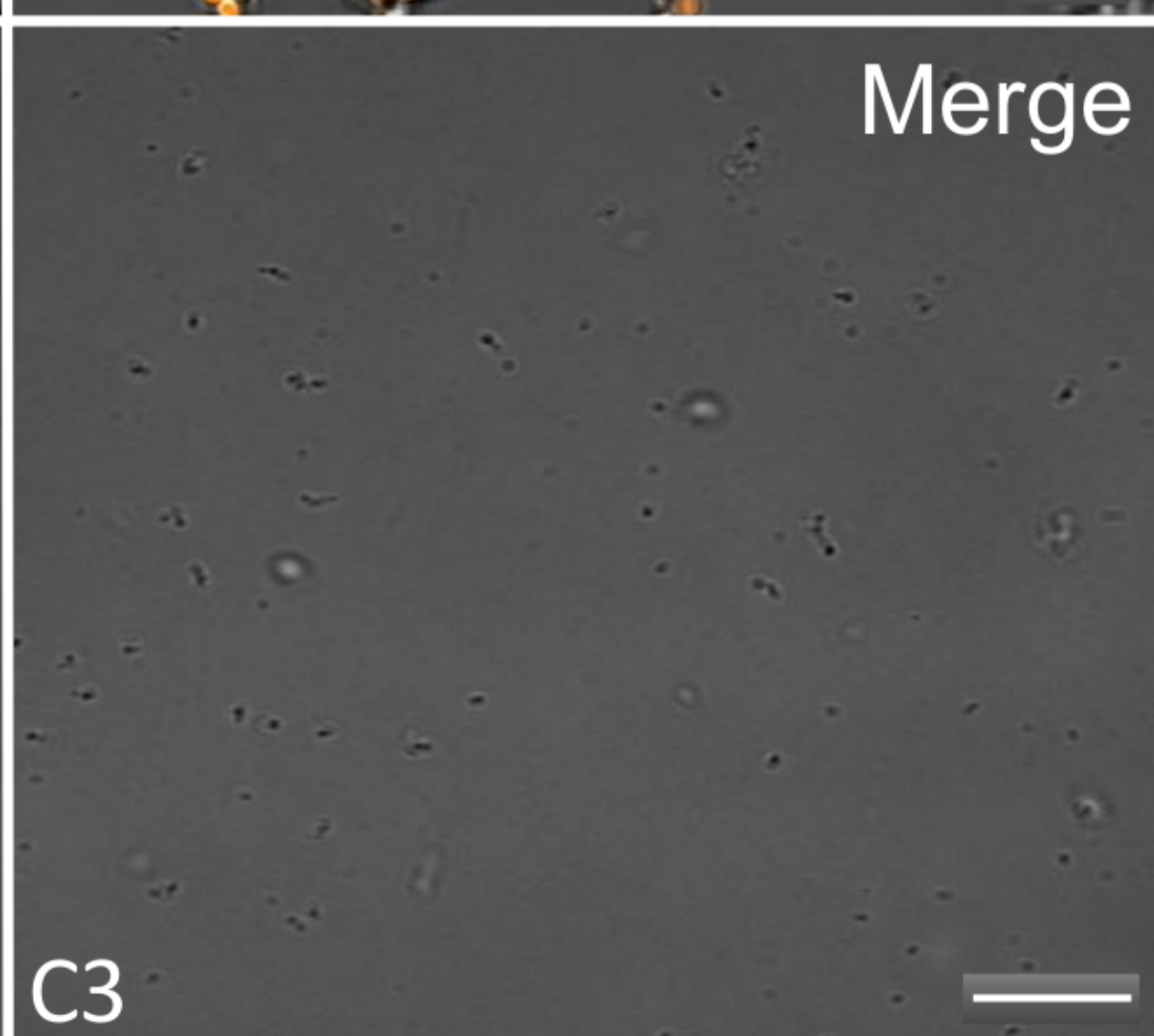

Supplement: Supplementary file 8 — Supplementary figure 2 [file 41396_2021_1117_MOESM8_ESM.pdf]

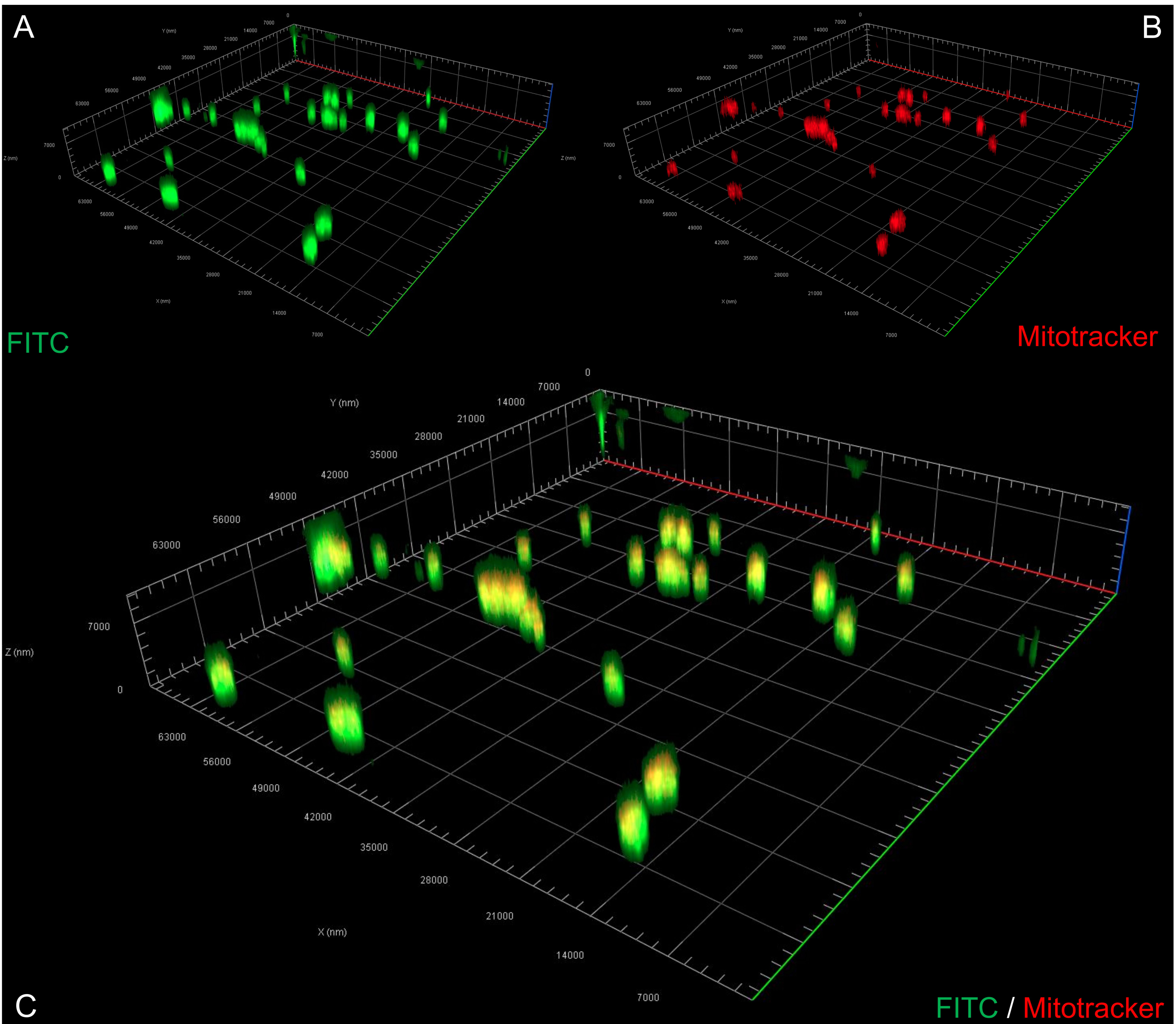

Supplement: Supplementary file 9 — Supplementary figure 3 [file 41396_2021_1117_MOESM9_ESM.pdf]

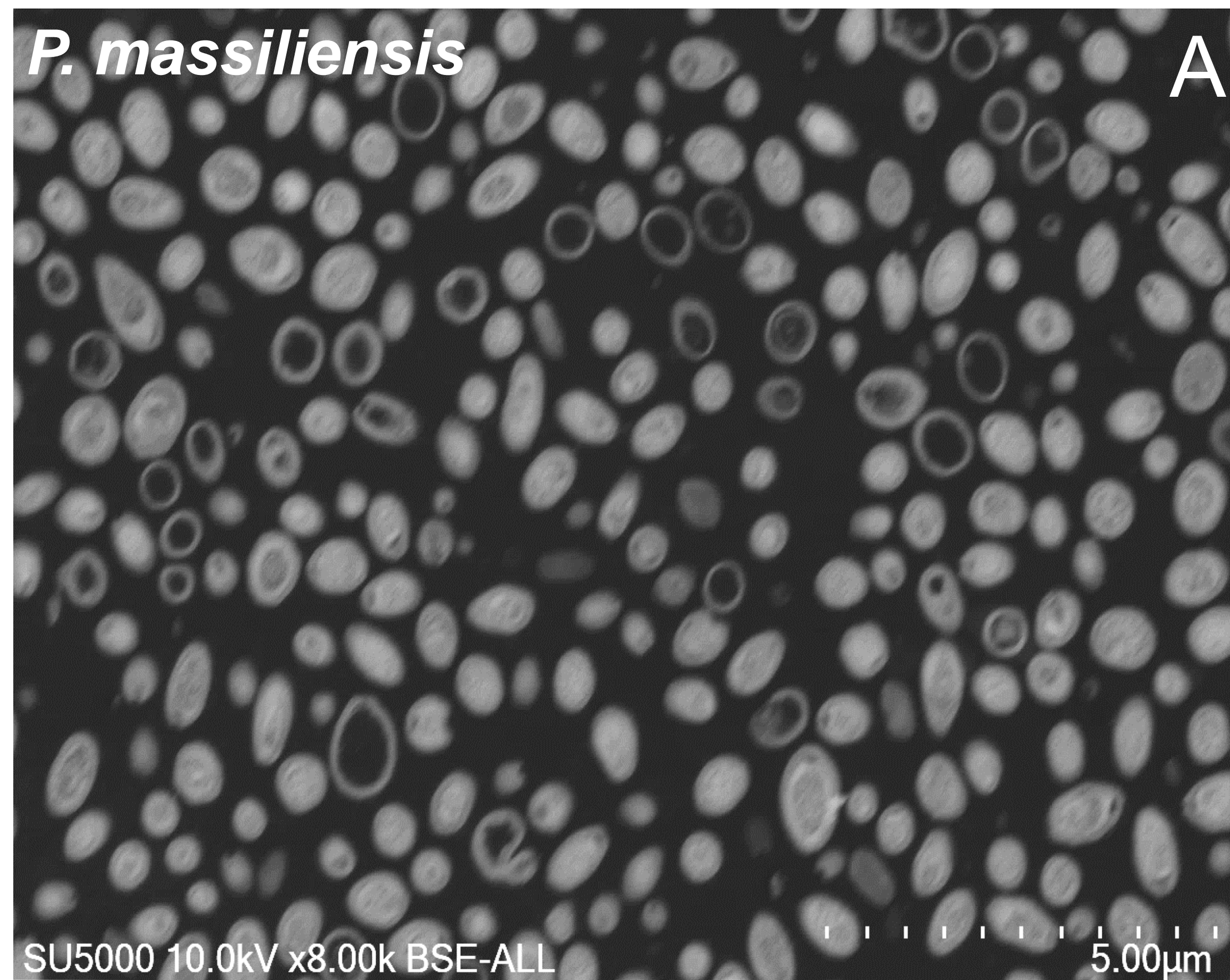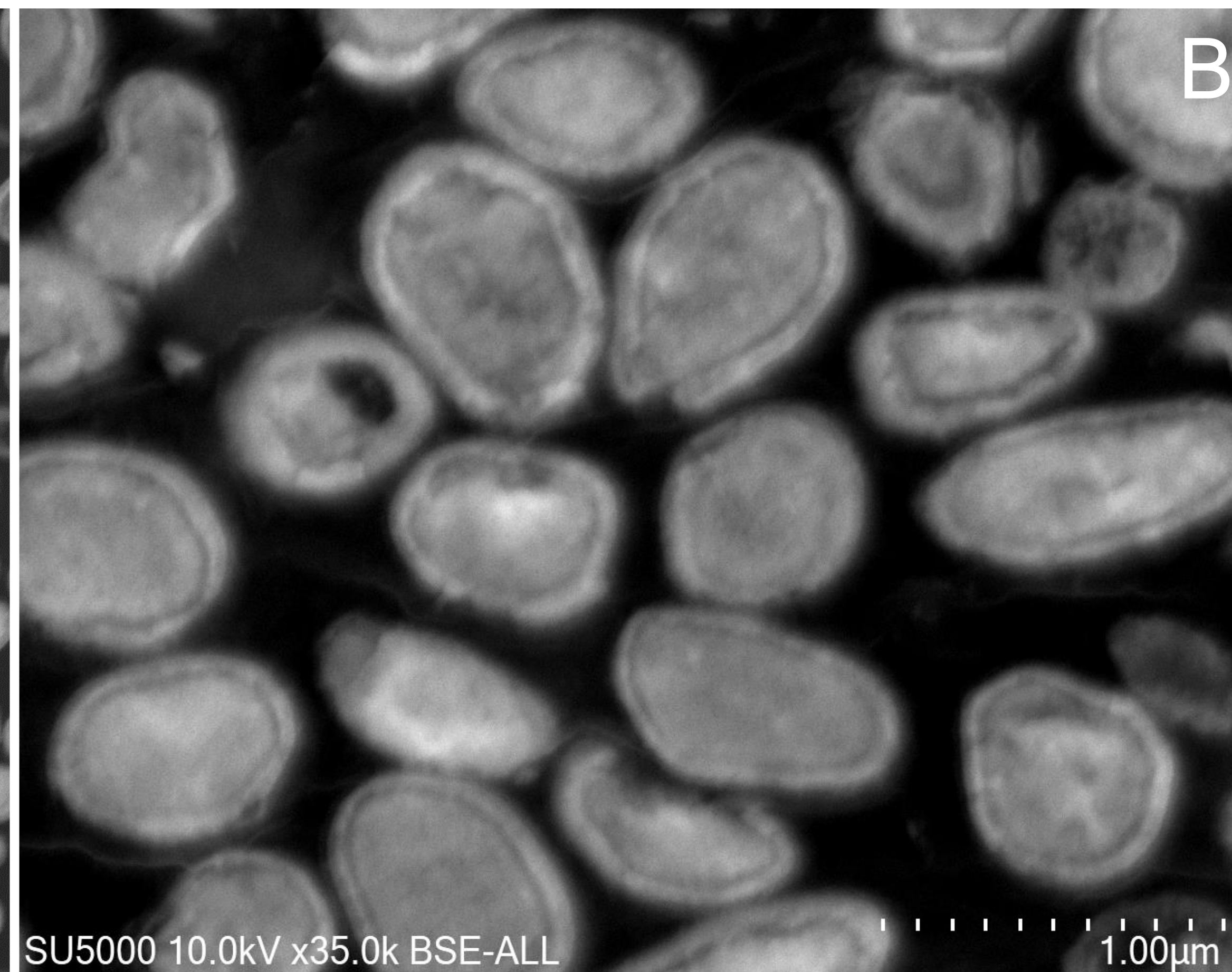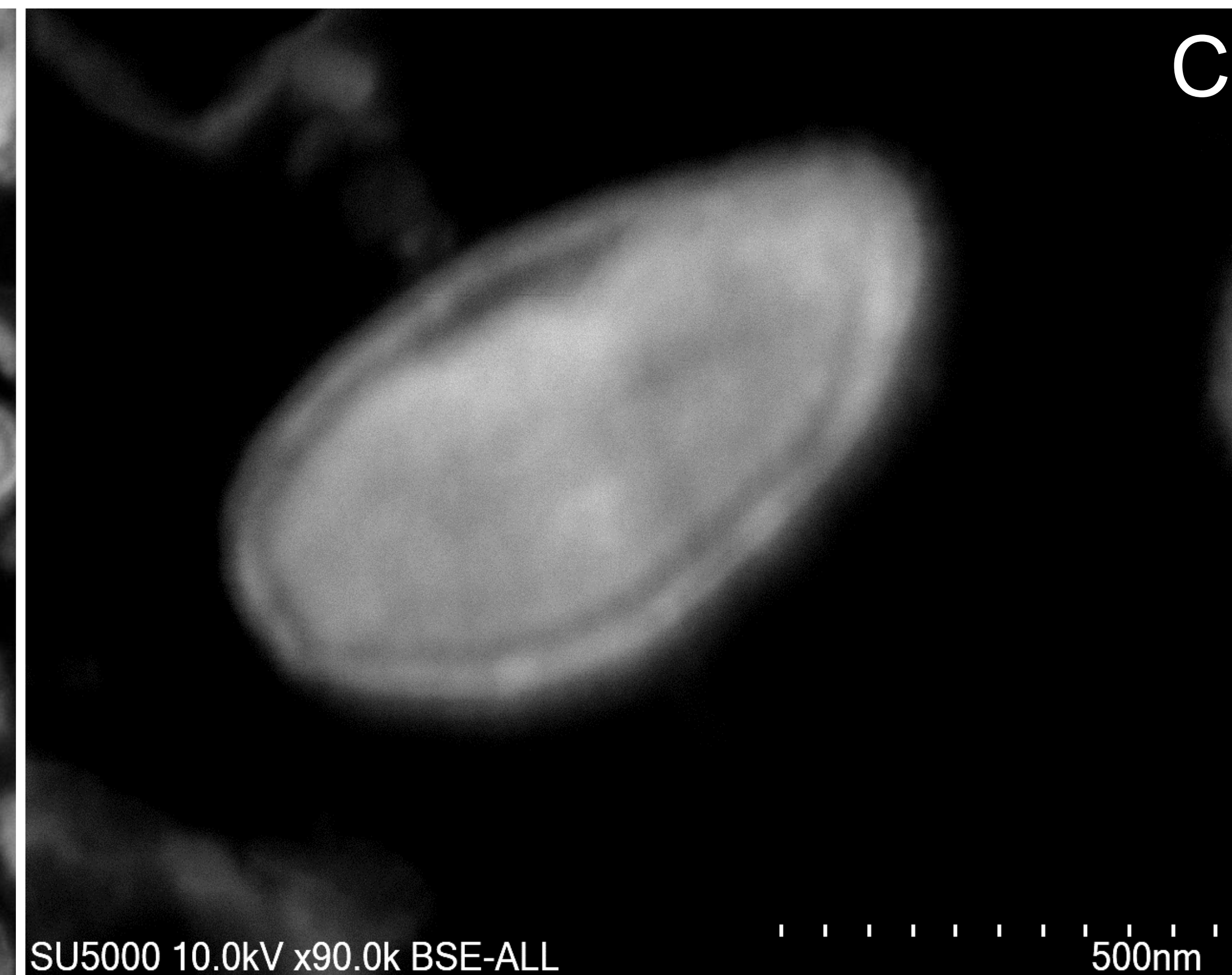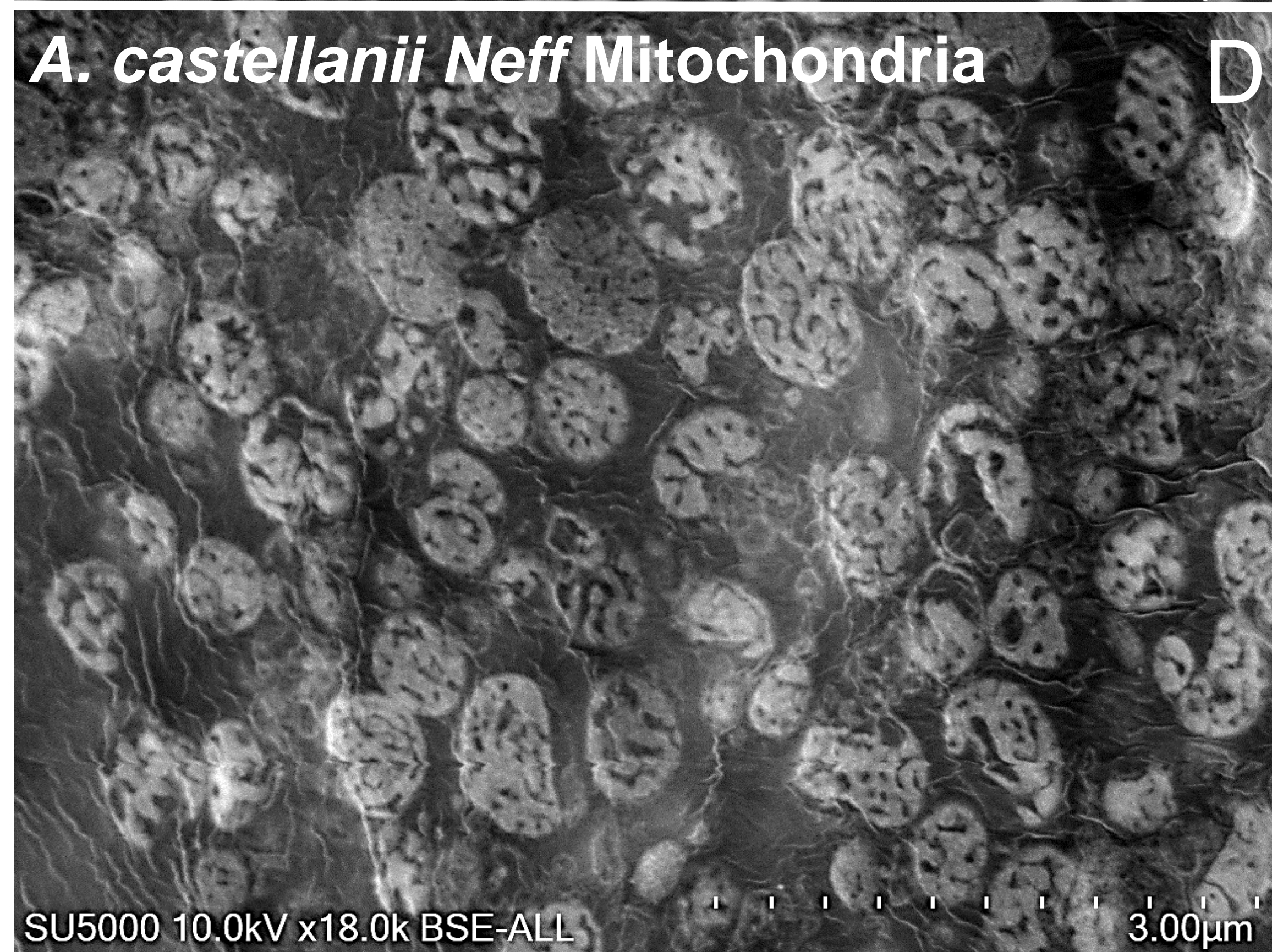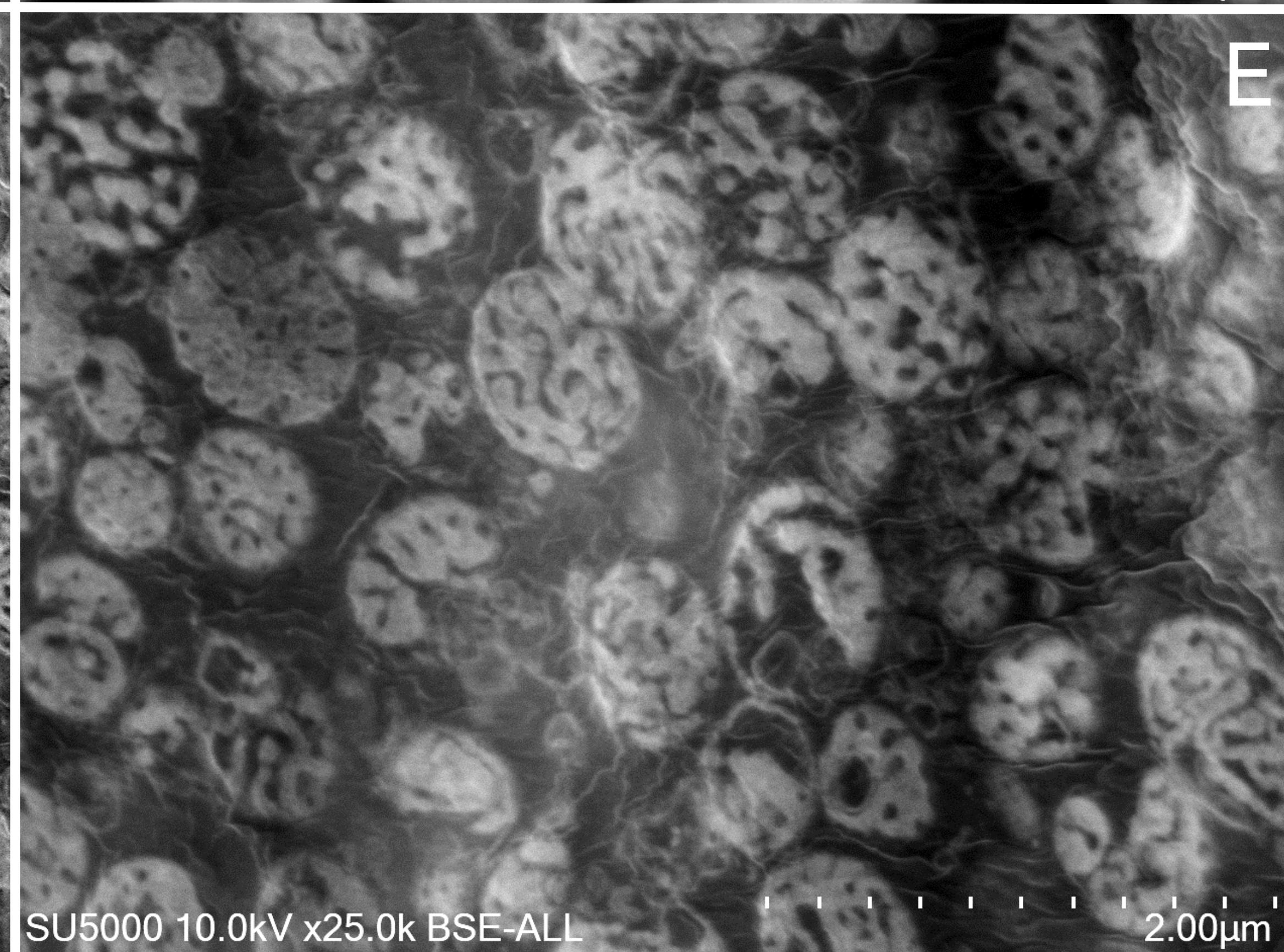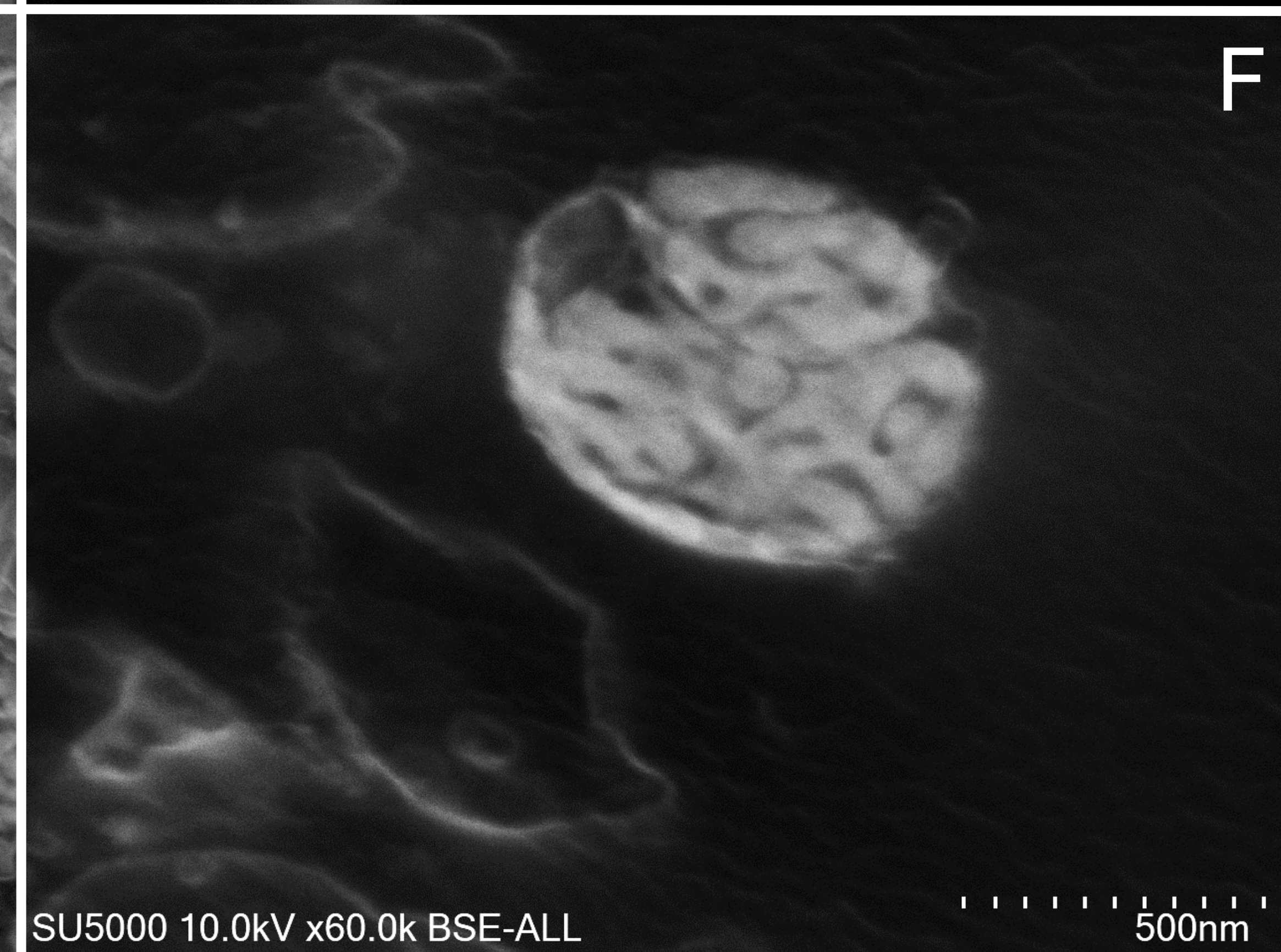

Supplement: Supplementary file 10 — Supplementary figure 4 [file 41396_2021_1117_MOESM10_ESM.pdf]

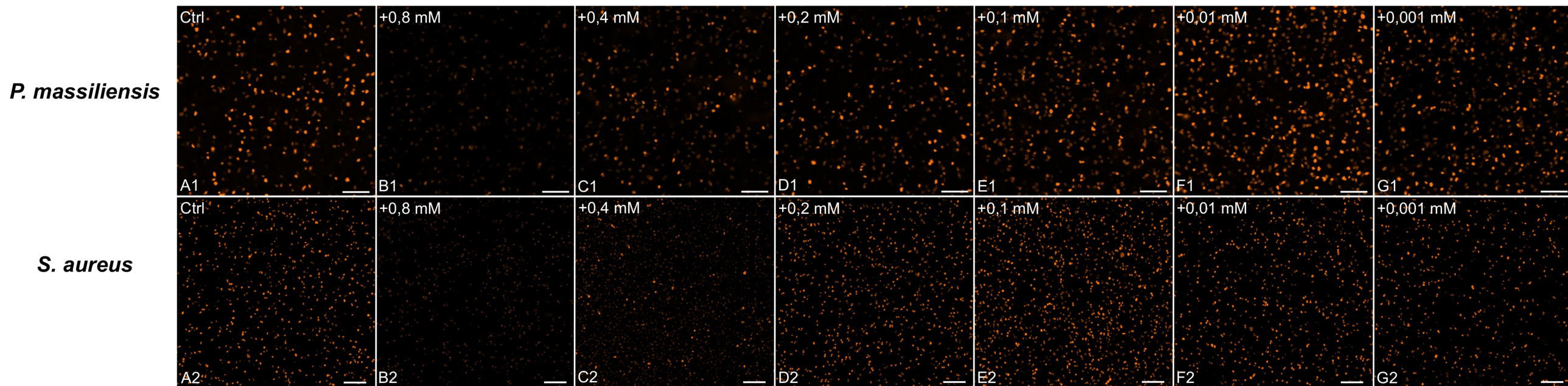

(I)

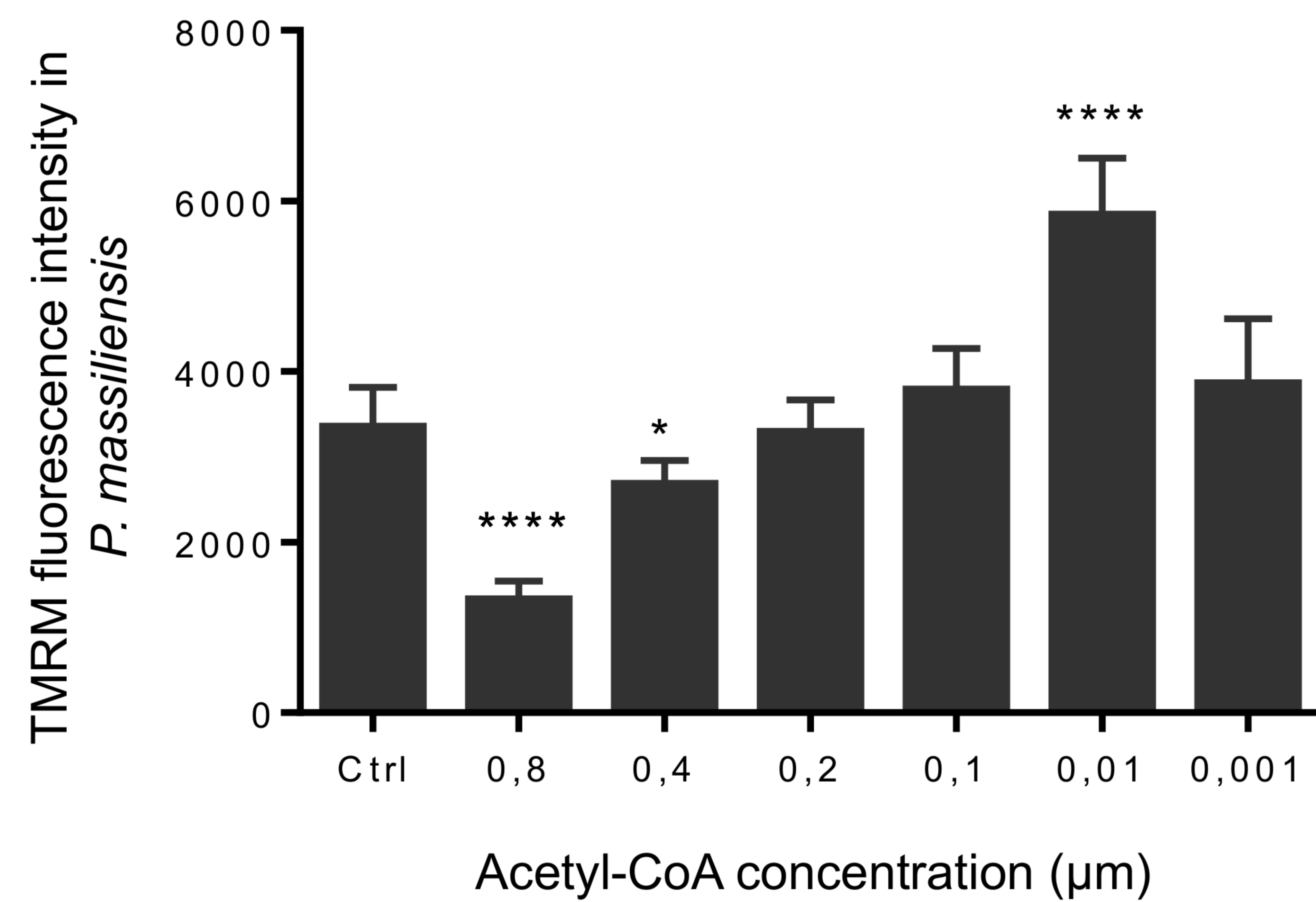

(II)

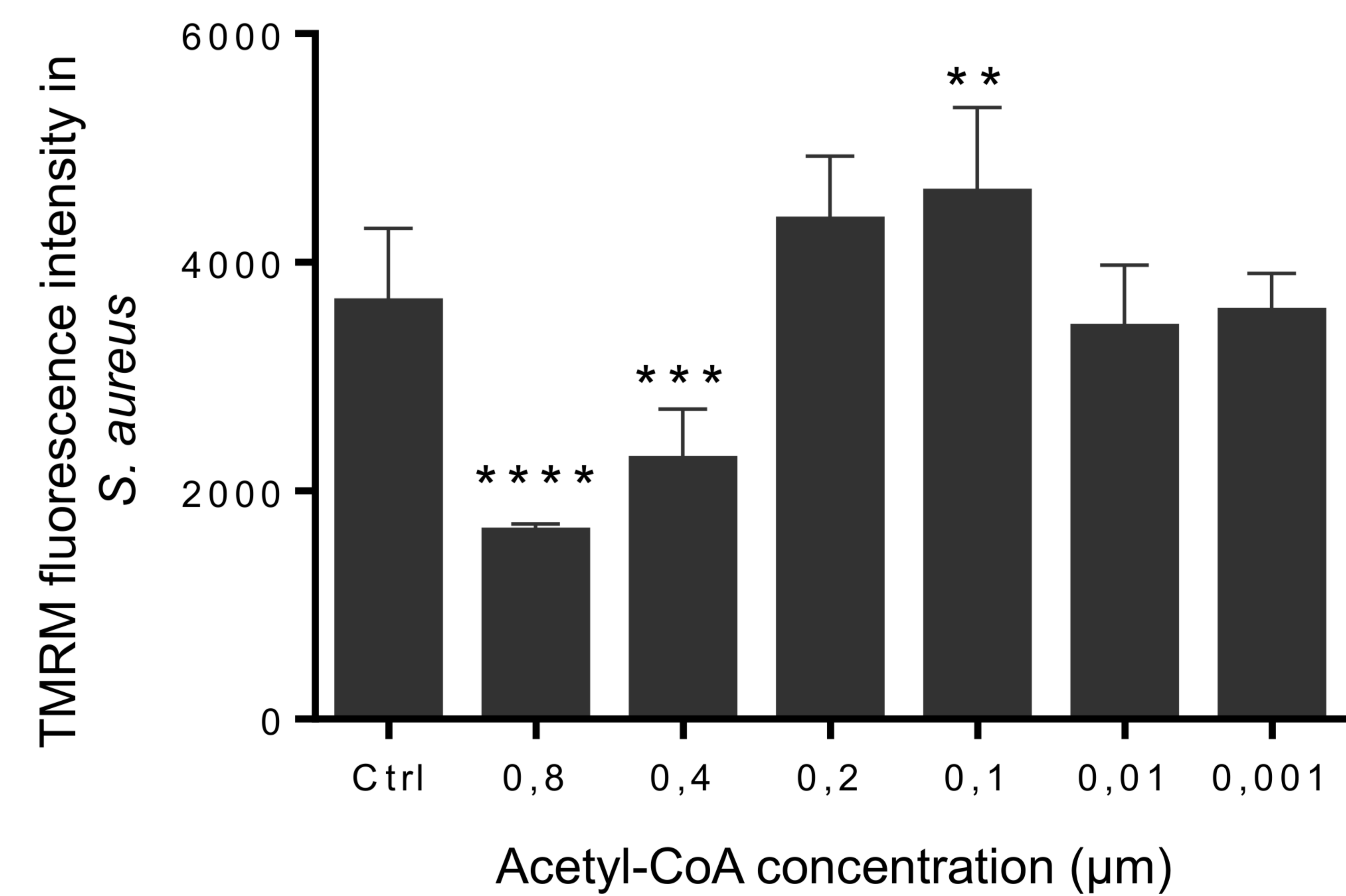

Supplement: Supplementary file 12 — Supplementary figure 6 [file 41396_2021_1117_MOESM12_ESM.pdf]

Supplementary figure 7

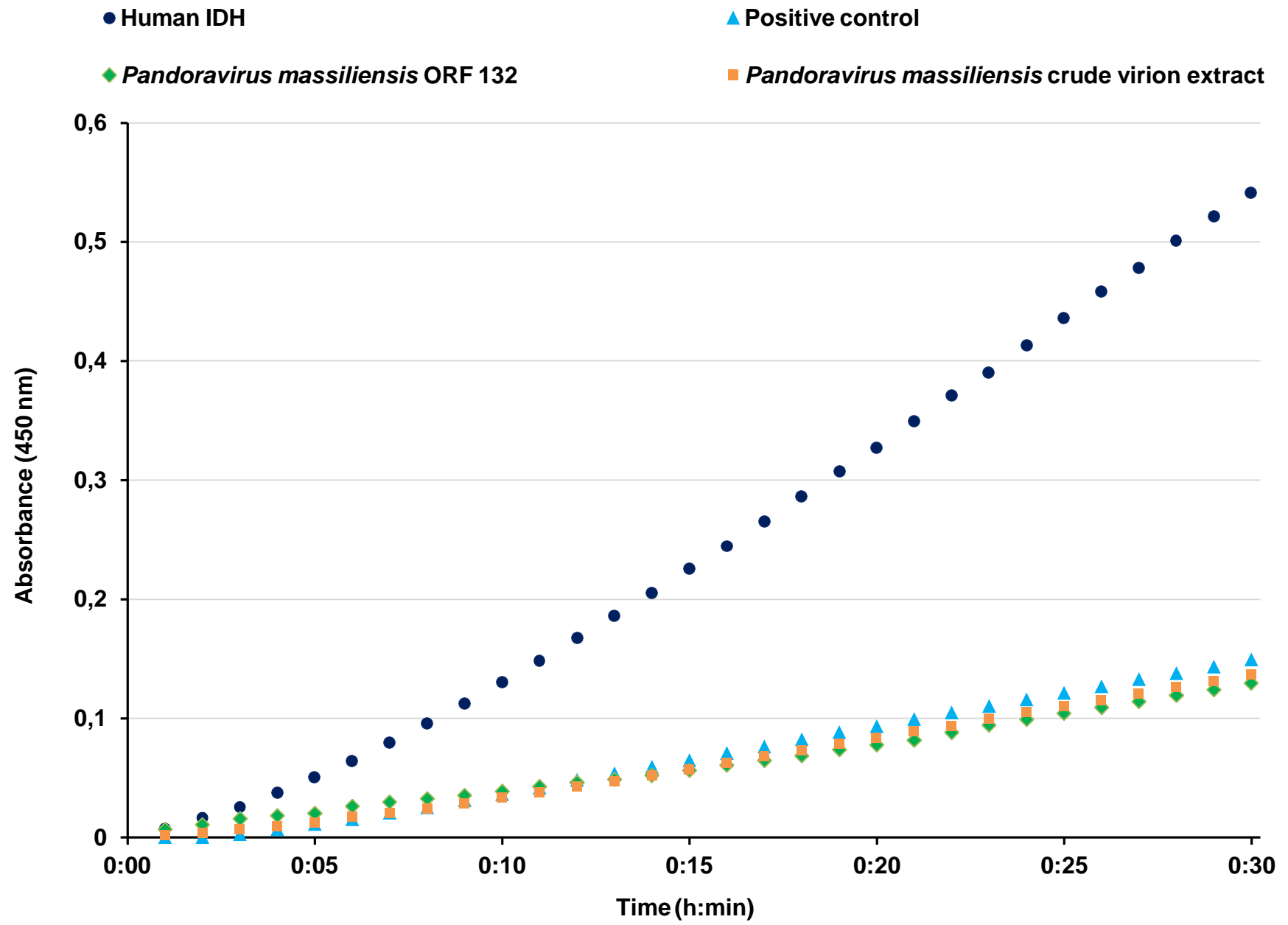

Supplement: Supplementary file 13 — Supplementary figure 7 [file 41396_2021_1117_MOESM13_ESM.pdf]

# Supplementary figure 8

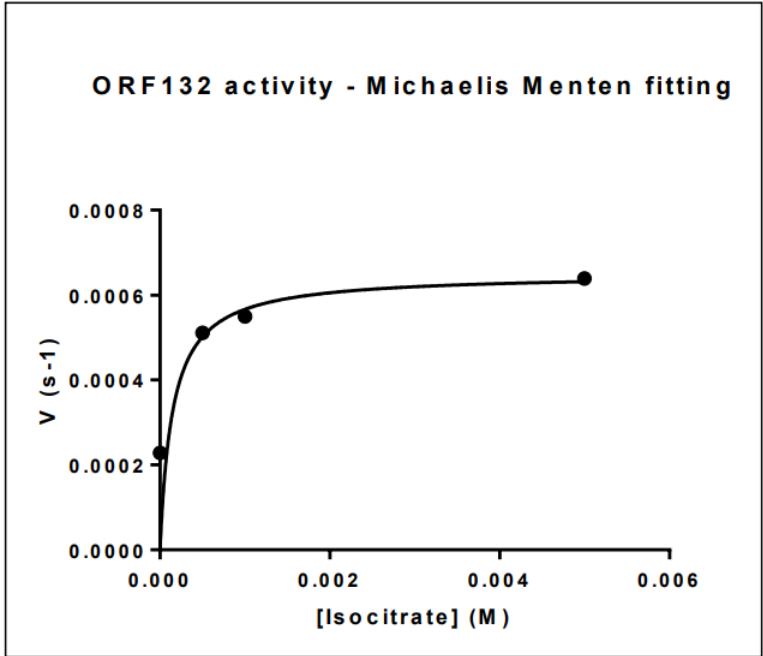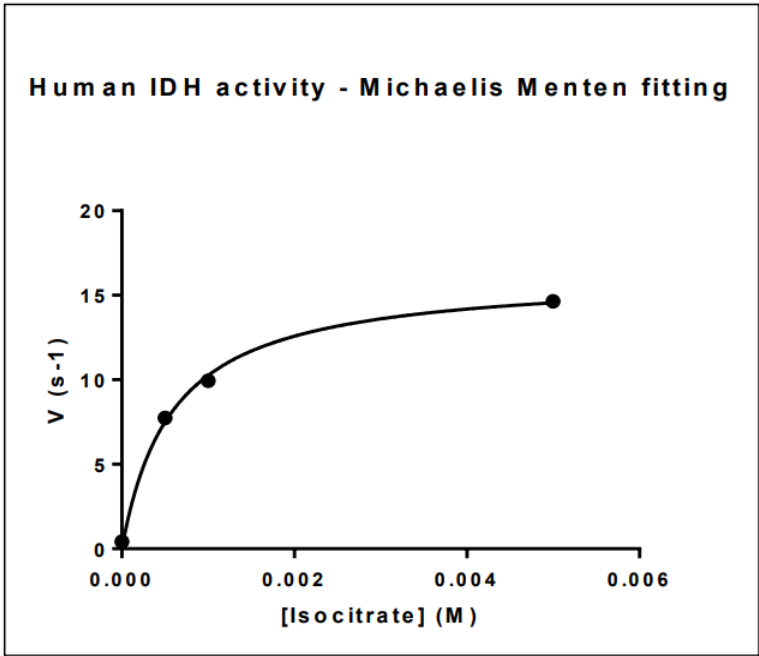

Supplement: Supplementary file 14 — Supplementary figure 8 [file 41396_2021_1117_MOESM14_ESM.pdf]
