# Supplementary material for: Incomplete tricarboxylic acid cycle and proton gradient in Pandoravirus massiliensis: is it still a virus?
Source: ISME J. 2021 Sep 23;16(3):695–704. doi: 10.1038/s41396-021-01117-3 (PMC8857278; doi:10.1038/s41396-021-01117-3)
Supplement: Supplementary file 6 [file 41396_2021_1117_MOESM6_ESM.docx]

Supplementary file 6: Detection of *P. massiliensis* predicted TCA ORFs by qRT-PCR at different time points.

| **ORFs** | **Predicted Enzyme** | **H0** | **H2** | **H4** | **H6** | **H8** | **H10** | **H12** | **H14** | **H16** |
| --- | --- | --- | --- | --- | --- | --- | --- | --- | --- | --- |
| **ORF595** | Acetyl CoA synthetase | NA | NA | NA | 34,1 | 31,7 | 31,9 | 29,4 | 28,2 | 26,5 |
| **ORF577** | Citrate synthetase | 32,6 | 33,2 | 31,2 | 29,1 | 28,7 | 29,3 | 28,9 | 27,7 | 26,3 |
| **ORF1245** | Aconitase | 33,0 | 32,9 | 31,2 | 28,0 | 28,3 | 29,6 | 29,1 | 25,7 | 23,8 |
| **ORF132** | Isocitrate dehydrogenase | 33,1 | 31,1 | 29,1 | 28,9 | 28,6 | 29,4 | 27,6 | 25,4 | 25,9 |
| **ORF864** | Isocitrate dehydrogenase | NA | NA | 33,2 | 32,5 | 31,9 | 33,5 | 28,1 | 26,4 | 25,1 |
| **ORF762** | α-ketoglutarate decarboxylase | NA | NA | NA | 31,8 | 36,3 | 31,9 | 29,9 | 24,8 | 26,2 |
| **ORF181** | Succinate dehydrogenase | 34,4 | 34,1 | 32,0 | 29,8 | 28,3 | 30,1 | 29,1 | 26,5 | 23,3 |
| **ORF206** | Fumarase | 32,6 | 29,4 | 26,9 | 26,6 | 26,5 | 27,7 | 25,3 | 26,8 | 21,9 |

Footnotes: The numbers in each case are the Ct obtained for qPCR. NA: No amplification.
