# Supplementary material for: Incomplete tricarboxylic acid cycle and proton gradient in Pandoravirus massiliensis: is it still a virus?
Source: ISME J. 2021 Sep 23;16(3):695–704. doi: 10.1038/s41396-021-01117-3 (PMC8857278; doi:10.1038/s41396-021-01117-3)
Supplement: Supplementary file 11 — Supplementary figure 5 [file 41396_2021_1117_MOESM11_ESM.pdf]

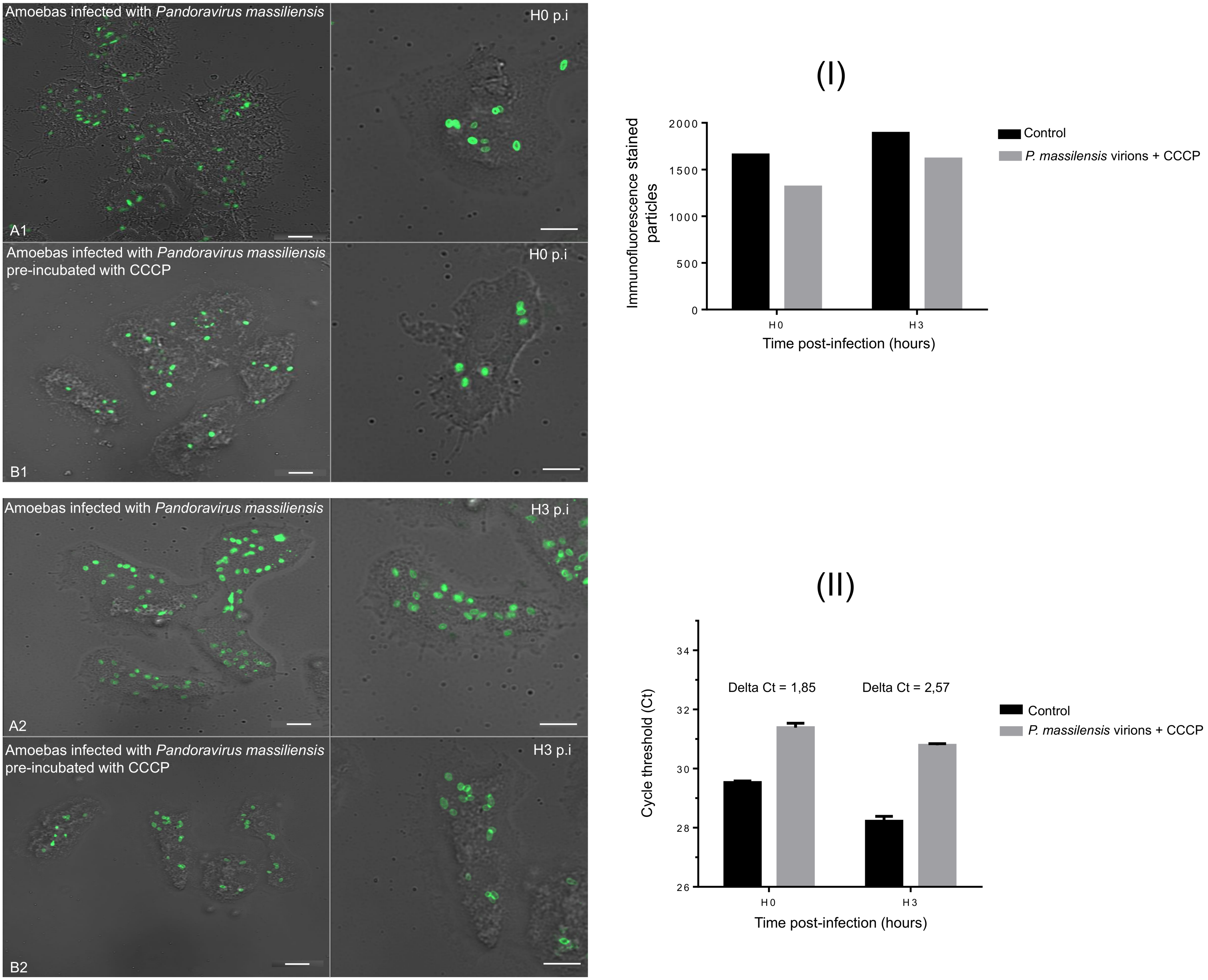
Amoebas infected with Pandoravirus massiliensis
H0 p.i
A1
Amoebas infected with Pandoravirus massiliensis pre-incubated with CCCP
H0 p.i
B1
Amoebas infected with Pandoravirus massiliensis
H3 p.i
A2
Amoebas infected with Pandoravirus massiliensis pre-incubated with CCCP
H3 p.i
B2
(I)
Immunofluorescence stained particles
2000
1500
1000
500
0
H0
H3
Time post-infection (hours)
Control
P. massilensis virions + CCCP
(II)
Cycle threshold (Ct)
34
32
30
28
26
Delta Ct = 1,85
Delta Ct = 2,57
H0
H3
Time post-infection (hours)
Control
P. massilensis virions + CCCP
