# Supplementary material for: Incomplete tricarboxylic acid cycle and proton gradient in Pandoravirus massiliensis: is it still a virus?
Source: ISME J. 2021 Sep 23;16(3):695–704. doi: 10.1038/s41396-021-01117-3 (PMC8857278; doi:10.1038/s41396-021-01117-3)
Supplement: Supplementary file 15 — Legends for supplementary files/figs [file 41396_2021_1117_MOESM15_ESM.docx]

**SUPPLEMENTARY FIGURE LEGENDS**

**Supplementary figure 1. Confocal imaging of MitoTracker staining of viral mature**

**particles of *P. massiliensis.*** **A**: Specific antibody-stained viral particles (FITC) (white arrows). B: MitoTracker Deep Red (red) incorporated into *P. massiliensis* particles (white arrow). C,D: Colocalization of the MitoTracker signal (red) with *P. massiliensis* virions marked by specific antibodies (white and blue arrow). The scales bar correspond to 2 µm.

**Supplementary figure 2. TMRM fluorescent staining of viral mature particles of *P.***

***massiliensis*** (A1-C1). Positive control consisting of *S. aureus* (A2-C2) and negative controls

consisting of cowpoxvirus (A3-C3) are shown. The scales bar correspond to 5 µm.

**Supplementary figure 3. 3D confocal microscope imaging of purified *P. massiliensis* particles stained with MitoTracker Deep Red.** **A**: Immunofluorescence signal-stained *P. massiliensis* (FITC). **B**: MitoTracker Deep Red (red) staining of *P. massiliensis* particles. **C**: 3D reconstruction image showing the co-localization of MitoTracker signal (red) with immunofluorescence signal of stained *P. massiliensis* (FITC).

**Supplementary figure 4. Scanning electron microscope (SEM) imaging of purified *P. massiliensis* virions and of amoebal isolated mitochondria.** **A**: Overview of purified *P. massiliensis* showing the absence of amoebal structure, including mitochondria, in the purified Pandoravirus sample. B: High-magnification SEM image of purified *P. massiliensis* **C**: SEM image of single *P. massiliensis* particle. D-E : SEM images of isolated amoebal mitochondria. F: Single amoebal mitochondria showing distinct morphological features compared to those of Pandoravirus particles.

**Supplementary figure 5. Assessment of the CCCP treatment effect on *P. massiliensis***

**infectivity.**

(A1-B2): Immunofluorescence confocal imaging of stained amoeba infected with *P.*

*massiliensis* particles preincubated with and without CCCP. (A1) Negative control: *P.*

*massiliensis* particles (green) in amoeba at H0 p.i. (B1) *P. massiliensis* + CCCP in amoeba at

H0 p.i. (A2) Negative control: *P. massiliensis* virions in the absence of CCCP (green) in

amoeba at H3 p.i. (B2) *P. massiliensis* particles (green) in amoeba at H3 p.i. (I): Estimation of

the number of stained particles of *P. massiliensis* particles without and with the highest

concentration of CCCP (400 µm) per/100 amoebas at H0, H3 p.i. (II): Representation of the

mean threshold cycle (Ct) of the qPCR experiments (triplicate) for isolated *P. massiliensis*

DNA before and after CCCP treatment according to the post-infection time from 0 to 3 h. The scales bar correspond to 5 µm.

**Supplementary figure 6. TMRM fluorescence intensity evaluation following acetyl CoA**

**treatment.** (A1-G1): Confocal imaging of TMRM staining following acetyl CoA treatment of P. massiliensis particles. A1: Control condition with untreated *P. massiliensis* particles. B1, G1: *P. massiliensis* virions treated with different concentrations of acetyl CoA. (A2-G2): Confocal imaging of TMRM staining after acetyl CoA treatment of the positive control (S. aureus). A2: Control experiment with untreated S. aureus. B2, G2: S. aureus treatment with a different concentration of acetyl CoA. (I): Estimation of the TMRM fluorescence intensity of *P.massiliensis* particles after acetyl CoA treatment. (II): Estimation of the TMRM fluorescence intensity of S. aureus after acetyl CoA treatment. The scales bar correspond to 10 µm.

**Supplementary figure 7. Evaluation of the enzymatic IDH activity of P. massiliensis**

**ORF132.**

**Supplementary figure 8. Michaelis-Menten plots of the IDH activity of the ORF132 of *P.***

***massiliensis* and of the human IDH.**

**SUPPLEMENTARY FILE LEGENDS**

**Supplementary file 1: qRT-PCR primers used in the present study of the predicted ORFs of *P. massiliensis.***

**Supplementary file 2: Results of the detailed bioinformatics analyses**

**Supplementary file 3: Alignment of the ORF132 with the COG0473 (Isocitrate / isopropylmalate dehydrogenase), its orthologs in other Pandoraviruses and 1408 sequences of isocitrate / isopropylmalate dehydrogenase from cellular organisms.** The first sheet of the excel tab shows the complete alignment of the whole sequences. The second sheet shows only the positions for the conserved amino-acid residues between the ORF132 of *P. massiliensis* and the COG0473.

**Supplementary file 4 : Maximum-likelihood phylogenetic tree based on the amino-acid sequences of the ORF132 of *P. massiliensis*, its orthologs in *P. neocaledonia* and *P. braziliensis* the COG0473 (isocitrate / isopropylmalate dehydrogenase) of *P. massiliensis* and 1408 representative hits from cellular organisms.** The alignment involves 565 amino-acids. Sequences from Pandoraviruses are indicated in red. Bootstrap values are given beside nodes in percent.

**Supplementary file 5: Pruned tree of the maximum-likelihood phylogenetic tree showed in the supplementary file 4.** Phylogeny based on 287 amino-acid sequences including the ORF132 of *P. massiliensis*, its orthologs in *P. neocaledonia* and *P. braziliensis,* indicated in red. The alignments involves 565 amino-acids. Bootstrap values are given beside nodes in percent.

**Supplementary file 6: Detection of *P. massiliensis* predicted TCA ORFs by qRT-PCR at different time points.**
